# Tuft cell IL-17RB restrains IL-25 bioavailability and reveals context-dependent ILC2 hypoproliferation

**DOI:** 10.1038/s41590-025-02104-y

**Published:** 2025-03-12

**Authors:** Xiaogang Feng, Tilde Andersson, Pascal Flüchter, Julia Gschwend, Ivan Berest, Julian L. Muff, Antonie Lechner, Aurelia Gondrand, Patrick Westermann, Nina Brander, Daniele Carchidi, Jeshua C. De Tenorio, Tianlang Pan, Ulrich Boehm, Christoph S. N. Klose, David Artis, Christoph B. Messner, Trese Leinders-Zufall, Frank Zufall, Christoph Schneider

**Affiliations:** 1 Institute of Physiology, University of Zurich, Zurich, Switzerland; 2 Institute of Molecular Health Sciences, ETH Zurich, Zurich, Switzerland; 3 Department of Pediatric Surgery, University Children’s Hospital of Basel, Basel, Switzerland; 4 Precision Proteomics Center, Swiss Institute of Allergy and Asthma Research (SIAF), University of Zurich, Davos, Switzerland; 5 Experimental Pharmacology, Center for Molecular Signaling (PZMS) and Center for Gender-Specific Biology and Medicine (CGBM), Saarland University, Homburg, Germany; 6 Department of Microbiology, Infectious Diseases and Immunology, Charité—Universitätsmedizin Berlin, corporate member of Freie Universität Berlin and Humboldt-Universität zu Berlin, Berlin, Germany; 7 Jill Roberts Institute for Research in Inflammatory Bowel Disease, Weill Cornell Medicine, Cornell University, New York, NY USA; 8 Friedman Center for Nutrition and Inflammation, Weill Cornell Medicine, Cornell University, New York, NY USA; 9 Allen Discovery Center for Neuroimmune Interactions, Weill Cornell Medicine, Cornell University, New York, NY USA; 10 Joan and Sanford I. Weill Department of Medicine, Weill Cornell Medicine, Cornell University, New York, NY USA; 11 Center for Integrative Physiology and Molecular Medicine, Saarland University, Homburg, Germany

**Keywords:** Mucosal immunology, Innate lymphoid cells

## Abstract

The tuft cell–group 2 innate lymphoid cell (ILC2) circuit orchestrates rapid type 2 responses upon detecting microbially derived succinate and luminal helminths. Our findings delineate key mechanistic steps involving IP3R2 engagement and Ca^2+^ flux, governing interleukin-25 (IL-25) production by tuft cells triggered by succinate detection. While IL-17RB has a pivotal intrinsic role in ILC2 activation, it exerts a regulatory function in tuft cells. Tuft cells exhibit constitutive *Il25* expression, placing them in an anticipatory state that facilitates rapid production of IL-25 protein for ILC2 activation. Tuft cell IL-17RB is crucial for restraining IL-25 bioavailability, preventing excessive tonic ILC2 stimulation due to basal *Il25* expression. Supraoptimal ILC2 stimulation by IL-25 resulting from tuft cell *Il17rb* deficiency or prolonged succinate exposure induces a state of hypoproliferation in ILC2s, also observed in chronic helminth infection. Our study offers critical insights into the regulatory dynamics of IL-25 in this circuit, highlighting the delicate tuning required for responses to diverse luminal states.

## Abstract (web summary)

Tuft cells constitutively express IL-25 to sustain ILC2 homeostasis in the intestine, but mechanisms driving IL-25 secretion have been unclear. Here, Feng et al. find that tuft cells express IL-17RB, which is required to regulate the bioavailability of IL-25 and to restrain the activation of ILC2s during homeostasis.

## Main

The single-layered epithelium of the small intestine separates luminal content from the underlying host tissue. In addition to their vital role in nutrient absorption, intestinal epithelial cells monitor the luminal status by detecting specific nutrients and microorganisms. These luminal signals can be sensed by distinct epithelial lineages and relayed to other cell types and tissues, leading to downstream responses that contribute to metabolic and immune regulation.

Chemosensory tuft cells are present in most mucosal epithelia, including the intestine, and have emerged as critical cells in processing information regarding luminal status^1^. In the small intestine, tuft cells detect eukaryotic parasites such as helminths and protists, as well as certain shifts in bacterial composition and associated metabolites^2^. Upon helminth infection, tuft cells produce interleukin-25 (IL-25) and cysteinyl leukotrienes, activating lamina propria-resident group 2 innate lymphoid cells (ILC2s)^3–6^. ILC2s respond by upregulating canonical type 2 cytokines, including IL-13, which promotes tuft and goblet cell differentiation. This tuft cell–ILC2 circuit is also activated by succinate, which can be produced by *Tritrichomonas* protists or bacteria, stimulating tuft cells expressing the cognate G-protein-coupled receptor (GPCR) succinate receptor 1 (SUCNR1)^7–9^. The acute succinate-mediated circuit activation depends on tuft cell-derived IL-25 for ILC2 activation, with cysteinyl leukotrienes being dispensable in that context^6–8^. Despite the constitutive expression of the *Il25* transcript in tuft cells^3^, the regulatory mechanisms governing IL-25 synthesis and release, for subsequent rapid ILC2 activation, remain unclear.

IL-25, previously known as IL-17E, is a member of the IL-17 cytokine family that contains the structurally related proteins IL-17A–IL-17F^10^. While best studied for IL-17A and IL-17F, this cytokine family is believed to engage canonical nuclear factor-κB (NF-κB) signaling downstream of ACT1 recruitment to a cytokine-specific multimeric receptor complex. The signaling-competent IL-25 receptor comprises two distinct chains, IL-17RB and IL-17RA^11^. Recent structural studies demonstrate that IL-25 binds specifically to IL-17RB units, allosterically initiating the formation of a ternary complex with the IL-17RA subunits, necessary for downstream signaling events^12^. ILC2s in the small intestinal lamina propria express high levels of IL-17RB^7,13^. Other immune and nonimmune cells were also reported to respond to IL-25, including dendritic cells and keratinocytes^14–16^. IL-25 is sufficient to induce type 2 cytokine expression in ILC2s, and this response is abolished in mice lacking *Il17rb* globally^17,18^. However, selective genetic ablation of IL-25 signaling in ILC2s, to demonstrate its cell-intrinsic role under physiologically relevant stimulation of the tuft cell–ILC2 circuit, has not been reported thus far. Notably, excessive homeostatic activation caused by IL-25 signaling in ILC2s is constrained by the ubiquitin-editing enzyme A20 (TNFAIP3)^7^, as well as the cytokine-inducible SH2-containing protein (CISH)^19^. In contrast, little is known about the tuft cell-intrinsic regulation of circuit activity. Although tuft cells constitutively express the machinery for the biosynthesis of IL-25 and cysteinyl leukotrienes, they do not strongly stimulate ILC2s in the absence of an agonistic signal^7,8^. Thus, the mechanistic basis for how tuft cells control effector molecules such as IL-25 remains to be defined.

In this study, we aimed to address important yet unanswered questions regarding the regulation of IL-25 and the cell type-specific functions of its receptor, IL-17RB. We found that tuft cells rapidly release IL-25 triggered by intracellular calcium, activating ILC2s through the expression of IL-17RB on the latter. Tuft cell-intrinsic IL-17RB expression provides an unexpected mechanism for dampening homeostatic IL-25 release, protecting against the induction of a hypoproliferative state in ILC2s caused by prolonged exposure to IL-25.

## Results

### Succinate-induced IL-25 acts through ILC2-intrinsic IL-17RB

**Fig. 1:**
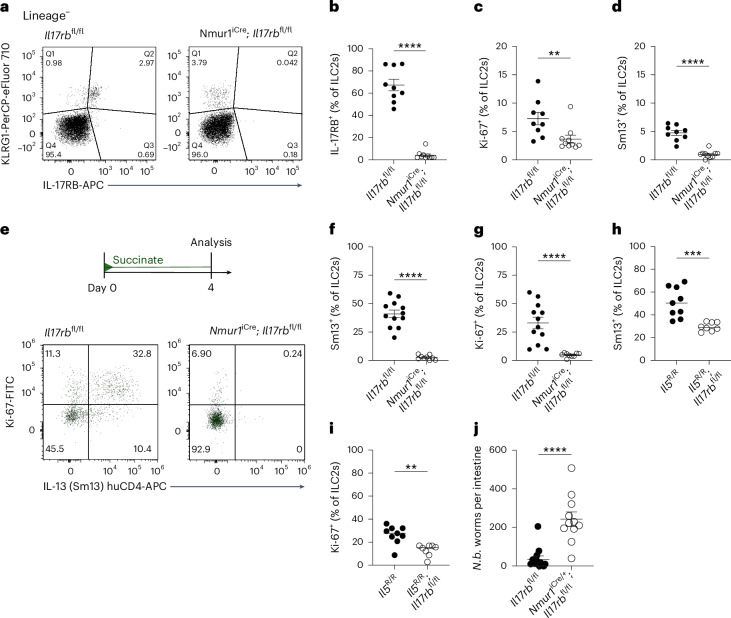
Succinate-induced IL-25 activates ILC2s in an ILC2-intrinsic, IL-17RB-dependent manner. **a**, Expression of IL-17RB and KLRG1 by lineage-negative (Lin^–^) cells. **b**–**d**, Frequencies of IL-17RB^+^ (**b**), Ki-67^+^ (**c**) and IL-13 (Sm13)^+^ (**d**) ILC2s quantified by flow cytometry (*n* = 9–10 mice). **e**, Experimental scheme (top) and expression of Ki-67 and the IL-13 (Sm13) reporter by ILC2s (bottom). **f**,**g**, Frequencies of IL-13 (Sm13)^+^ (**f**) and Ki-67^+^ (**g**) ILC2s quantified by flow cytometry (*n* = 9–12 mice). **h**,**i**, Percentage of small intestinal IL-13 (Sm13)^+^ (**h**) and Ki-67^+^ (**i**) ILC2s quantified by flow cytometry from *Il5*^R/R^ and *Il5*^R/R^; *Il17rb*^fl/fl^ mice treated with succinate for 4 days (*n* = 8–9 mice). **j**, *N. brasiliensis* (*N.b.*) worm burden in the small intestine on day 9 after infection (*n* = 11 mice). Data are indicated as mean or mean ± s.e.m. (**b**–**d**, **f**–**j**). Data were analyzed using the Mann–Whitney test (**b**–**d**, **f**, **g**, **j**) or a two-tailed unpaired *t* test (**h**, **i**). **P = 0.01–0.001; ***P = 0.001–0.0001; ****P < 0.0001.

**Extended Data Fig. 1:**
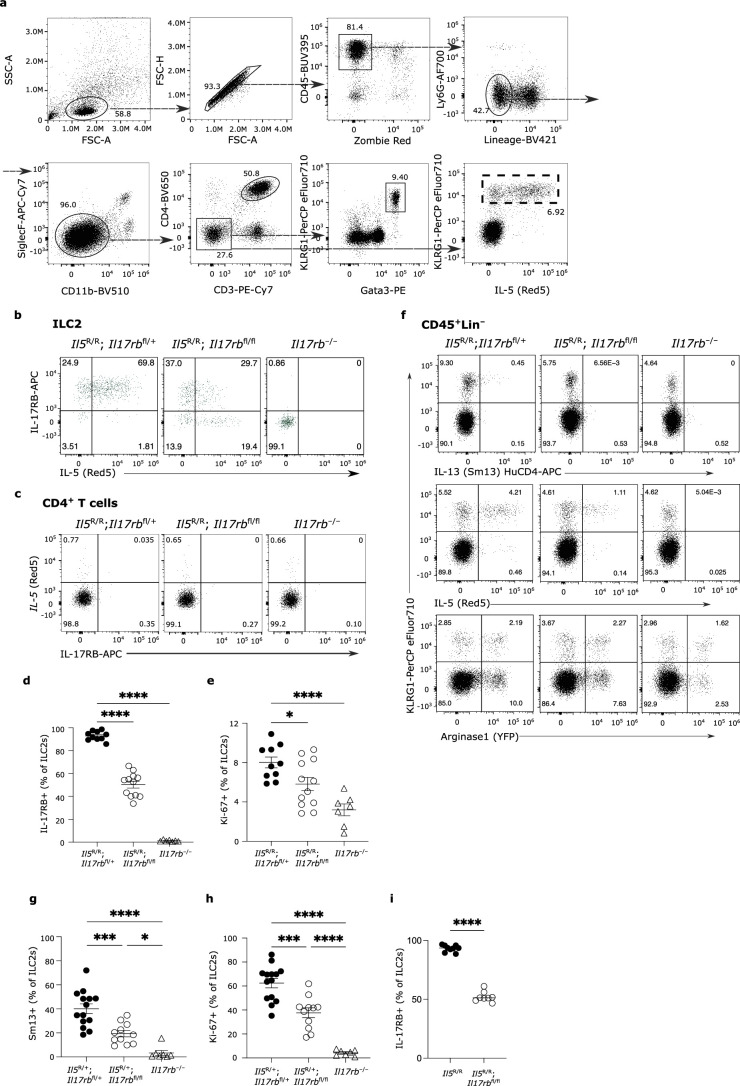
ILC2-intrinsic requirement for IL-17RB in homeostatic and succinate-induced ILC2 activation. **a**, Flow cytometry gating of small intestinal ILC2s, throughout the paper defined as either Gata3^+^KLRG1^+^ (fixed panel, filled line), or KLRG1^+^ (live panel, dashed line). **b**, Expression of IL-5 and IL-17RB by SI ILC2 cells from naïve *Il5*^R/R^;*Il17rb*^fl/+^, *Il5*^R/R^;*Il17rb*^fl/fl^, and *Il5*^+/+^;*Il17rb*^–/–^ mice. *Il5*^R^ allele: *Il5*^tdTomato-Cre^, known as “Red5”. **c**, Red5 or IL-17RB expression by CD3^+^CD4^+^ cells, gated as in a). **d**,**e**, Frequencies of IL-17RB^+^ (d) and Ki-67^+^ (e) ILC2s were quantified by flow cytometry (n = 7–12 mice). **f**, Expression of KLRG1 and *Arg1*^YFP^, IL-5 (Red5), and IL-13 (Sm13) reporters by Lin^–^ cells. **g**,**h**, Frequencies of IL-13 (Sm13)^+^ (g) and Ki-67^+^ (h) ILC2s were quantified by flow cytometry from *Il5*^R/+^;*Il17rb*^fl/+^, *Il5*^R/+^;*Il17rb*^fl/fl^ and *Il17rb*^–/–^ mice which were treated with succinate for 4 days (n = 7–14 mice). **i**, Frequencies of IL-17RB^+^ ILC2s were quantified by flow cytometry from *Il5*^R/R^ and *Il5*^R/R^;*Il17rb*^fl/fl^ mice treated with succinate for 4 days (n = 8–9 mice). Data mean (i) or mean ± s.e.m (d,e,g,h) is indicated. Data was analyzed using Ordinary one-way ANOVA (d,e,g,h) or two-tailed unpaired t test (i). *P 0.01–0.05; **, P 0.01–0.001; ***, P 0.001–0.0001;****, P < 0.0001; ns, P ≥ 0.05.

To assess the ILC2-intrinsic requirement for IL-17RB, we generated mice with an *Il17rb* deficiency in *Il5*-expressing cells (*Il5*^R^; *Il17rb*^fl^) by crossing *Il17rb*^fl^ mice with ‘YRS’ mice, which express reporter alleles for the ILC2 signature markers arginase-1 (‘Yarg’ allele; *Arg1*^YFP^), IL-5 (‘Red5’ allele; *Il5*^tdTomato-Cre^, referred to as *Il5*^R^) and IL-13 (‘Smart13’ (Sm13) allele; *Il13*^huCD4^). In *Il5*^R/R^; *Il17rb*^fl/+^ control mice, we observed the expression of the IL-5 reporter and IL-17RB in most of the small intestinal ILC2s (Extended Data Fig. 1a,b), consistent with prior literature^7^. CD4^+^ T cells from the same tissue did not show substantial expression of IL-17RB or the IL-5 reporter (Extended Data Fig. 1c). ILC2s from *Il5*^R/R^; *Il17rb*^fl/fl^ mice showed reduced IL-17RB staining, with residual expression present in IL-5^−/low^ ILC2s (Extended Data Fig. 1b,d). IL-17RB expression was completely absent in ILC2s from *Il17rb*^–/–^ mice (Extended Data Fig. 1b,d). To confirm the ILC2-intrinsic requirement for IL-17RB further, we crossed *Il17rb*^fl^ with *Nmur1*^iCre^ mice, which efficiently and specifically target ILC2s^20,21^. *Nmur1*^iCre^; *Il17rb*^fl/fl^ mice showed near-complete *Il17rb* deletion in ILC2s compared to their littermate controls (Fig. 1a,b). We observed reduced Ki-67 expression in *Il17rb*-deficient ILC2s, suggesting tonic effects of IL-25 signaling in ILC2s (Fig. 1c and Extended Data Fig. 1e). In these *Tritrichomonas*-free SOPF (specific and opportunistic pathogen-free) mice, some ILC2s expressed *Arg1*^YFP^, and overall, IL-13 expression was minimal, in agreement with prior studies reporting low baseline ILC2 activation in the absence of luminal stimuli for the tuft cell–ILC2 circuit (Fig. 1d and Extended Data Fig. 1f)^7^.

To address the role of IL-17RB in ILC2s upon circuit activation, we treated mice with the luminal tuft cell agonist succinate in drinking water. After 4 days, control mice exhibited readily upregulated expression of IL-13 and Ki-67, which was abrogated in *Nmur1*^iCre^; *Il17rb*^fl/fl^ mice (Fig. 1e–g). IL-13 and Ki-67 expression was also significantly reduced in *Il5*^R/R^; *Il17rb*^fl/fl^ and *Il5*^R/+^; *Il17rb*^fl/fl^ mice compared to their controls, although to a lesser extent, reflecting incomplete *Il17rb* deletion (Fig. 1h,i and Extended Data Fig. 1g–i). These results establish that IL-25 directly stimulates ILC2s through their IL-17RB following the activation of tuft cells with succinate. Additionally, *Nmur1*^iCre^; *Il17rb*^fl/fl^ mice showed a significant impairment in clearing an infection with the helminth *Nippostrongylus brasiliensis* (Fig. 1j).

### Succinate activates tuft cells through IP3R2 and Ca^2+^ activity

**Fig. 2:**
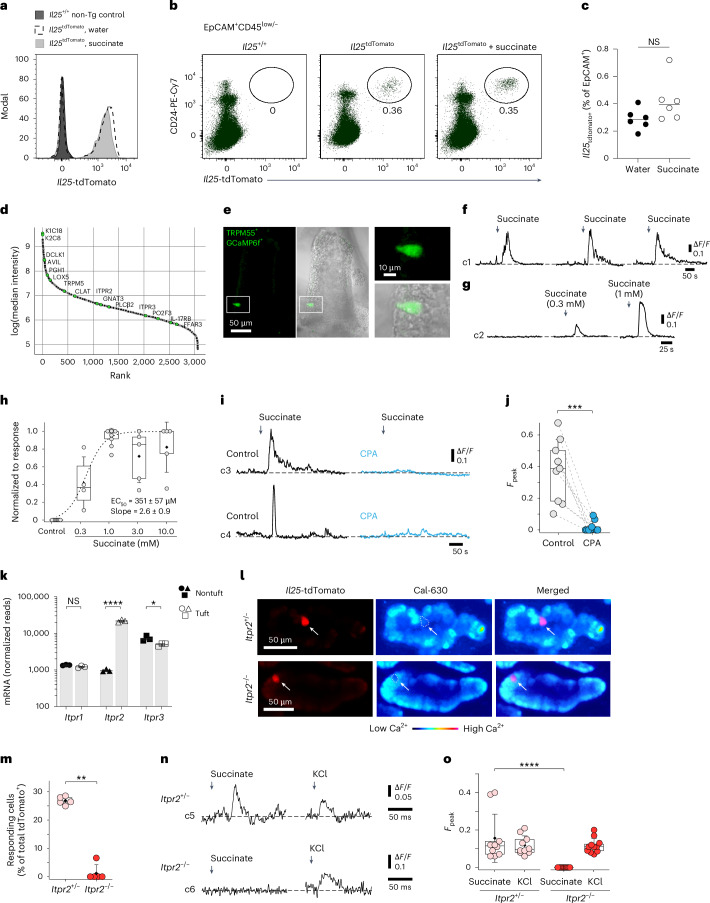
Succinate-induced tuft cell activation depends on IP3R2-mediated cytosolic Ca^2+^ activity. **a**–**c**, *Il25*^tdTomato^ reporter mice were treated with succinate or regular drinking water. The small intestine was analyzed on day 4. **a**, *Il25*^tdTomato^ expression in CD24^+^EpCAM^+^ tuft cells determined by flow cytometry and compared to tuft cells from a reporter-negative mouse. **b**, Frequencies of *Il25*^tdTomato+^CD24^+^ tuft cells gated on EpCAM^+^CD45^low/–^ cells. **c**, Percentage of *Il25*^tdTomato+^ cells (*n* = 6 mice). **d**, Proteins detected by liquid chromatography–mass spectrometry (LC–MS) in tuft cells, ranked by relative abundance. Tuft cell-specific marker proteins are labeled with their UniProt identifiers (*n* = 5 mice). **e**–**j**, Tuft cell Ca^2+^ activity in *Trpm5*^Cre^; *R26*^GCaMP6f^ mice. **e**, Tuft cells (green) in ex vivo scraped villus preparations; a GCaMP6f-expressing tuft cell (white box) is magnified. **f**, Reproducible Ca^2+^ response time courses of a tuft cell induced by 1 mM succinate. **g**, Ca^2+^ response time courses of a tuft cell to increasing succinate concentrations and no succinate control (*n* = 4–10 tuft cells, villus preparations from two to three mice). **h**, Dose–response curve of Ca^2+^ peak responses normalized to the maximum response of a tuft cell. **i**, Example traces of tuft cells stimulated with 1 mM succinate before (control) and after 10 μM CPA (blue). **j**, Peak Ca^2+^ responses from preparations as in **h**. *F*_peak_ values in nine random tuft cells (villus preparations from three mice) are shown. **k**, *Itpr1*, *Itpr2* and *Itpr3* mRNA expression in tuft and nontuft cells from the small intestine (published dataset: Nadjsombati et al.^8^; *n* = 3 mice). **l**–**o**, Tuft cell Ca^2+^ activity with the Cal-630 indicator dye in villus preparations from *Il25*^tdTomato^ *Itpr2*^+/–^ and *Itpr2*^–/–^ mice. **l**, *Il25*^tdTomato^-expressing tuft cells (red) after Cal-630 indicator loading. **m**, Percentage of succinate-responding tuft cells (*n* = 107–117 tuft cells from five mice per genotype; Mann–Whitney test). **n**,**o**, Examples of Ca^2+^ response time courses (**n**) and quantification of peak response (**o**) to 1 mM succinate and 60 mM KCl (positive control) in tuft cells from *Il25*^tdTomato^ *Itpr2*^+/–^ and *Itpr2*^–/–^ mice (*F*_peak_ values of 10–11 independent tuft cell measurements from three mice per genotype). Data were analyzed using the Mann–Whitney test (**c**, **h**, **m**, **o**), a paired *t* test (**j**) or a two-tailed unpaired *t* test (**k**). Box plots display the interquartile (25–75%) ranges, median (line) and mean (black square) values with whiskers indicating s.d. values. *P = 0.01–0.05; **P = 0.01–0.001; ***P = 0.001–0.0001; ****P < 0.0001; NS (no significance), *P* ≥ 0.05.

**Extended Data Fig. 2:**
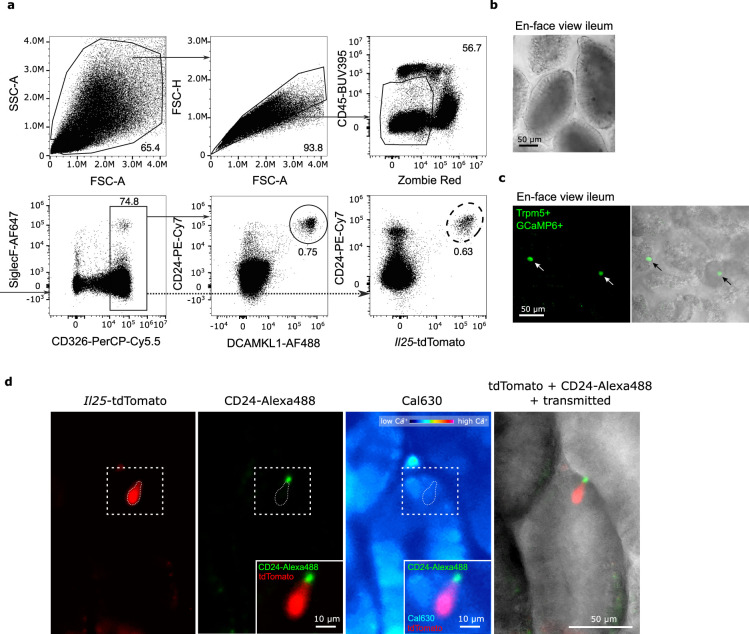
Visualization of *Il25* expression and Ca^2+^ signaling in tuft cells. **a**, Flow cytometry gating of small intestinal tuft cells, throughout the paper defined as either DCLK1^+^CD24^+^ (fixed panel, filled line), or *Il25*^tdTomato+^CD24^+^ double positive (live panel, dashed line). **b**, Tuft cell Ca^2+^ activity of the ileum was imaged with an *ex vivo* whole mount (en-face view of the ileum) preparation from *Trpm5*^Cre^;*R26*^GCaMP6f^ mice. **c**, Tuft cells (*green*) can be identified based on *Trpm5* promoter-dependent expression of GCaMP6f (*arrows*). **d**, Tuft cell Ca^2+^ activity of the ileum was imaged with an *ex vivo* whole mount preparation from *Il25*^tdTomato^ (Flare25) mice using a Cal630 Ca^2+^ indicator dye. Tuft cells (white box) can be identified based on their distinct *Il25* (*red*) and CD24 (*green*) (*arrows*).

Intrigued by the ability of the succinate–tuft cell axis to stimulate ILC2s rapidly through IL-25 signaling, we further explored the mechanism underlying this response. Comparing the expression of an *Il25*^tdTomato^ transcriptional reporter allele in tuft cells of mice treated with water or succinate revealed no significant changes in *Il25* transcript levels or the frequency of *Il25*^tdTomato+^ cells (Fig. 2a–c and Extended Data Fig. 2a). These results agree with prior findings that demonstrated constitutive expression of *Il25* mRNA in tuft cells across various tissues^3^. Proteome analysis of sorted tuft cells showed high expression of tuft cell signature proteins, including doublecortin-like kinase 1 (DCLK1), transient receptor potential cation channel subfamily M member 5 (TRPM5) and arachidonate 5-lipoxygenase (ALOX5), whereas IL-25 was not detected (Fig. 2d).

Tuft cells engage canonical taste transduction signaling components when triggered by luminal agonists such as succinate, including the Ca^2+^-activated monovalent cation channel TRPM5 (refs. ^7–9^). We hypothesized that increasing cytosolic Ca^2+^ is a critical step in tuft cells for their biosynthesis of IL-25. To test this, we visualized Ca^2+^ activity in isolated villi using the genetically encoded fast Ca^2+^ sensor GCaMP6f, expressed in TRPM5^+^ tuft cells, as recently published for tracheal tuft cells^22,23^. In these *Trpm5*^Cre^; *R26*^GCaMP6f^ mice, temporally and spatially resolved Ca^2+^ signals can be recorded by confocal imaging in tuft cells in their native cellular environment in the small intestinal villus epithelium (Fig. 2e and Extended Data Fig. 2b,c). Exposure to succinate produced reproducible and concentration-dependent transient Ca^2+^ elevations in TRPM5^+^ tuft cells (Fig. 2f–h and Supplementary Video 1). The succinate-induced Ca^2+^ response was abolished when SERCA (sarcoplasmic/endoplasmic reticulum calcium adenosine triphosphatase) was inhibited with cyclopiazonic acid (CPA), indicating that Ca^2+^ release from intracellular Ca^2+^ stores is required for these responses (Fig. 2i,j). Type II taste bud cells release the second messenger ATP in a process that depends on the calcium channel inositol 1,4,5-trisphosphate receptor 3 (IP3R3)^24^. However, analysis of a published RNA-sequencing dataset and our tuft cell proteome data revealed that tuft cells differentially express high levels of the *Itpr2* transcript and its encoded protein, IP3R2 (also known as ITPR2), compared to nontuft epithelial cells, whereas *Itpr3* and *Itpr1* transcripts are expressed at lower and comparable levels to those in other epithelial cells (Fig. 2d,k). Using the Cal-630 Ca^2+^ indicator dye, we measured Ca^2+^ responses in tuft cells from *Il25*^tdTomato^; *Itpr2*^+/–^ and *Il25*^tdTomato^; *Itpr2*^–/–^ mice (Fig. 2l and Extended Data Fig. 2d). This analysis revealed a strongly impaired response to succinate in IP3R2-deficient tuft cells (Fig. 2m–o). Overall, our data suggest that IL-25 is controlled post-transcriptionally through IP3R2-regulated cytosolic Ca^2+^ activity.

### IP3R2 triggers succinate-induced IL-25 production in tuft cells

**Fig. 3:**
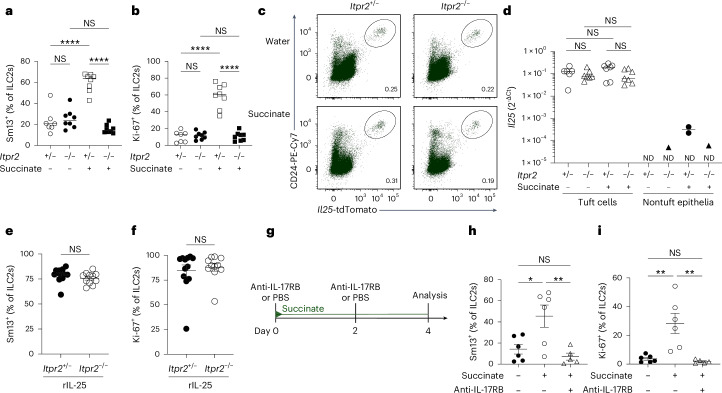
Succinate-elicited IL-25 production in tuft cells is triggered by IP3R2. **a**,**b**, Frequencies of small intestinal IL-13 (Sm13)^+^ (**a**) and Ki-67^+^ (**b**) ILC2s quantified by flow cytometry from *Il25*^tdTomato^; *Itpr2*^–/–^ and *Il25*^tdTomato^; *Itpr2*^+/–^ littermate mice treated with succinate for 4 days (*n* = 7–8 mice). **c**, Flow cytometry analysis of *Il25*^tdTomato^ (Flare25) reporter and CD24 expression, gated on EpCAM^+^ cells. **d**, *Il25* mRNA expression in tuft and nontuft epithelial cells sorted by fluorescence-activated cell sorting (FACS) from the proximal small intestine (*n* = 6–7 mice; ND, not detected in at least one sample). **e**,**f**, Frequencies of IL-13 (Sm13)^+^ (**e**) and Ki-67^+^ (**f**) ILC2s quantified by flow cytometry from *Il25*^tdTomato^; *Itpr2*^–/–^ and *Il25*^tdTomato^; *Itpr2*^+/–^ mice treated with 1 μg rIL-25 on two consecutive days (*n* = 11–12 mice). **g**, Mice expressing the IL-13 (Sm13) reporter were treated with succinate for 4 days and injected with PBS or an anti-IL-17RB blocking antibody on days 0 and 2. **h**,**i**, Frequencies of IL-13 (Sm13)^+^ (**h**) and Ki-67^+^ (**i**) ILC2s quantified by flow cytometry (*n* = 5–6 mice). Data are indicated as mean or mean ± s.e.m (**a**, **b**, **d**–**f**, **h**–**i**). Data were analyzed using a two-way analysis of variance (ANOVA) (**a**, **b**), a one-way ANOVA (**h**, **i**) or the Mann–Whitney test (**e**, **f**). *P = 0.01–0.05; **P = 0.01–0.001; ****P < 0.0001; NS, *P* ≥ 0.05.

**Extended Data Fig. 3:**
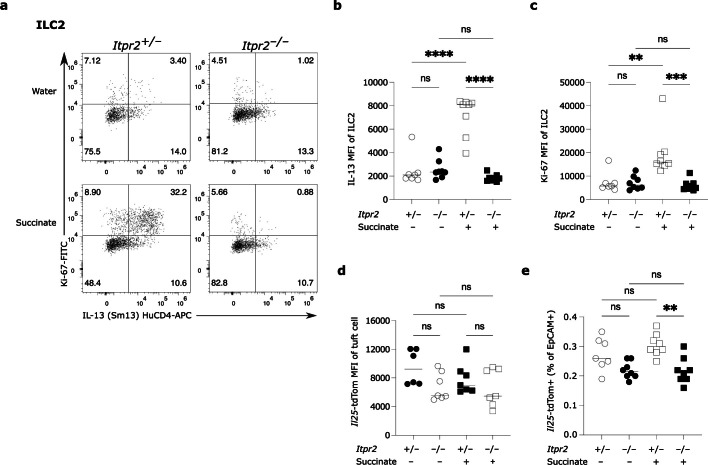
Succinate-induced ILC2 activation is abolished in *Itpr2*^–/–^ mice. **a**, Flow cytometry analysis of Ki-67 and IL-13 (Sm13)-reporter expression by ILC2s from *Itpr2*^*+/–*^ and *Itpr2*^*+/–*^ mice treated for 4 days with succinate. **b**-**d**, The MFI of IL-13 (Sm13) (b) and Ki-67 (c) expression in ILC2s, and of the *Il25*^tdTomato^ reporter in tuft cells (d) was quantified (n = 7–8 mice). **e**, Frequency of *Il25*^tdTomato+^ cells (n = 7–8 mice). Data median is indicated (b-e). Data was analyzed using two-way ANOVA(b-e). **, P 0.01–0.001; ***, P 0.001–0.0001; ****, P < 0.0001; ns, P ≥ 0.05.

To assess in vivo whether IP3R2-mediated Ca^2+^ activity controls post-transcriptionally regulated IL-25 production following succinate receptor stimulation, we treated *Itpr2*^–/–^ mice with succinate in drinking water. Indeed, succinate-induced expression of IL-13 and Ki-67 by ILC2s was abolished in *Itpr2*^–/–^ mice compared to the *Itpr2*^+/–^ littermate controls (Fig. 3a,b and Extended Data Fig. 3a–c). Notably, the percentage of tuft cells did not significantly differ from that before stimulation, and both the *Il25*^tdTomato^ reporter signal and the qPCR quantification showed comparable levels of *Il25* transcript expression (Fig. 3c,d and Extended Data Fig. 3d,e). When we bypassed the activation of tuft cells by directly injecting recombinant IL-25 (rIL-25), ILC2s of *Itpr2*^–/–^ mice responded similarly to those of *Itpr2*^+/–^ littermates, demonstrating that IP3R2 is not required in ILC2s for their stimulation by IL-25 (Fig. 3e,f). To address whether IL-25 protein is indeed released and binds to IL-17RB to activate ILC2s following tuft cell stimulation with succinate, we injected wild-type mice with an anti-IL-17RB blocking antibody^25^ during succinate treatment (Fig. 3g). This acute blockade of IL-17RB potently prevented the activation of ILC2s (Fig. 3h,i). Altogether, these results demonstrate that an IP3R2-dependent cytosolic Ca^2+^ signaling axis mediates the post-transcriptional control of IL-25, triggering its production and release upon tuft cell stimulation with succinate.

### Tuft cell IL-17RB restrains ILC2 activation and homeostatic circuit activity

**Fig. 4:**
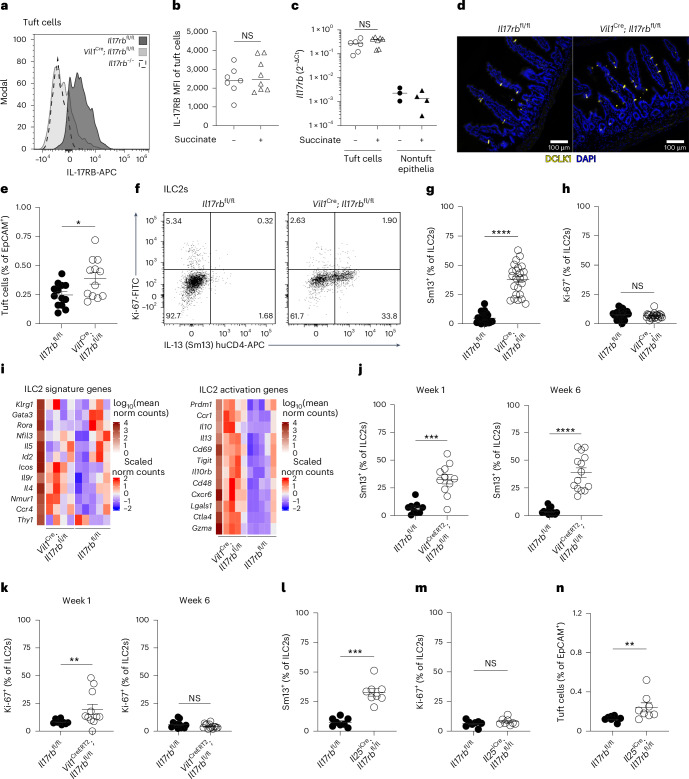
Tuft cell IL-17RB restrains homeostatic circuit activation. **a**, Expression of IL-17RB in small intestinal tuft cells from *Vil1*^Cre^; *Il17rb*^fl/fl^, *Il17rb*^fl/fl^ and *Il17rb*^–/–^ mice quantified by flow cytometry. **b**, Mean fluorescence intensity (MFI) of IL-17RB expression in tuft cells quantified by flow cytometry from *Il25*^tdTomato^ reporter mice treated with or without succinate for 4 days (*n* = 7–8 mice). **c**, *Il17rb* mRNA expression in tuft and nontuft epithelial cells FACSorted from the proximal small intestine of *Il25*^tdTomato^ reporter mice treated with or without succinate for 4 days (*n* = 6–7 mice). **d**, Representative immunofluorescence images of DCLK1 (yellow) in small intestines from naive *Vil1*^Cre^; *Il17rb*^fl/fl^ and *Il17rb*^fl/fl^ mice. **e**, Quantification of tuft cells by flow cytometry (*n* = 12–13 mice). **f**, Flow cytometry analysis of Ki-67 and IL-13 (Sm13) reporter expression by ILC2s. **g**,**h**, Frequencies of IL-13 (Sm13)^+^ (**g**) and Ki-67^+^ (**h**) ILC2s quantified by flow cytometry (*n* = 23–24 mice). **i**, Heatmaps showing the relative expression of ILC2 signature genes (left) and genes associated with ILC2 activation (right), generated from bulk RNA sequencing of FACSorted ILC2s (CD45^+^Lin^–^KLRG1^+^) from the small intestines of *Vil1*^Cre^; *Il17rb*^fl/fl^ and *Il17rb*^fl/fl^ mice (*n* = 4–5 samples). **j**,**k**, Frequencies of small intestinal IL-13 (Sm13)^+^ (**j**) and Ki-67^+^ (**k**) ILC2s quantified by flow cytometry from *Vil1*^CreERT2^; *Il17rb*^fl/fl^ and *Il17rb*^fl/fl^ mice 1 or 6 weeks after tamoxifen treatment (*n* = 9–14 mice). **l**–**n**, Frequencies of small intestinal IL-13 (Sm13)^+^ ILC2s (**l**), Ki-67^+^ ILC2s (**m**) and tuft cells (**n**) quantified by flow cytometry from *Il25*^iCre/+^; *Il17rb*^fl/fl^ and *Il17rb*^fl/fl^ mice (*n* = 8 mice). Data are indicated as median (**b**, **c**) or mean ± s.e.m (**e**, **g**, **h**, **j**, **k**, **l**–**n**). Data were analyzed using the Mann–Whitney test (**b**, **c**, **e**, **g**, **h**, **j**, **k**, **l**–**n**). *P = 0.01–0.05; **P = 0.01–0.001; ***P = 0.001–0.0001; ****P < 0.0001; NS, *P* ≥ 0.05.

**Extended Data Fig. 4:**
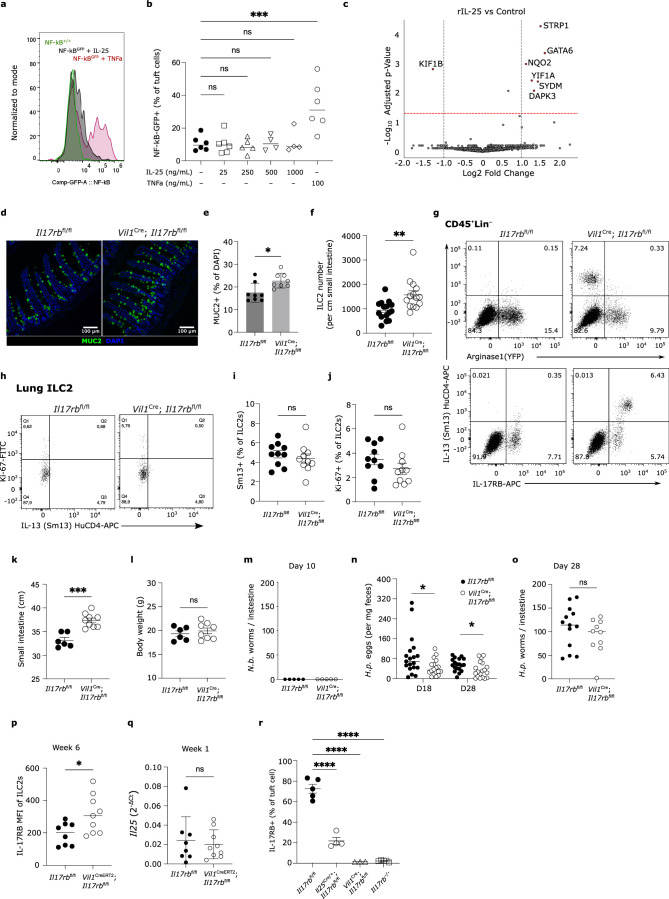
Tuft cell IL-17RB restrains homeostatic circuit activation. **a**, GFP reporter signal in tuft cells analyzed by flow cytometry of SI organoids from NF-κB^GFP^ activity reporter mice treated with indicated cytokines for 24 h. **b**, Frequency of NF-kB^GFP+^ tuft cells (n = 1–2 technical replicates on 2 biological replicates). **c**, Tuft cell proteomics analysis (n = 3–5 mice). Volcano plot showing differentially expressed proteins in tuft cells from IL-25 treated mice compared to controls. Each point represents a detected protein. Proteins with significant expression changes are labelled with their UniProt identifiers. **d**, IF of MUC2 in SI (scale bars, 100 μm). **e**, Muc2 area relative to DAPI area per imaging field (n = 8 mice). **f**, SI ILC2s numbers (n = 14–15 mice). **g**, Expression of IL-13 (Sm13) and *Arg1*^YFP^ reporters or IL-17RB by CD45^+^Lin^–^ cells. **h**, Ki-67 and IL-13 (Sm13)-reporter expression by lung ILC2s. **i**,**j**, Frequencies of IL-13 (Sm13)^+^ (i) and Ki-67^+^ (j) ILC2s, quantified by flow cytometry (n = 10 mice). **k**,**l**, SI length (k) and body weight (l) of the indicated mice (n = 6–9 mice). **m**, Day 10 SI *N. brasiliensis* worm burden (n = 5 mice). **n**, Fecal egg burden on day 8 and 28 post *H. polygyrus* infection (n = 19 mice). **o**, Worm burden 28 days after infection (n = 10–13 mice). **p**, IL-17RB MFI of ILC2s of *Vil1*^CreERT2^;*Il17rb*^fl/fl^ mice, 6 weeks post tamoxifen (n = 8–9 mice). **q**, *Il25* mRNA expression in tuft cells from *Vil1*^CreERT2^;*Il17rb*^fl/fl^ and *Il17rb*^fl/fl^ mice 1 week post tamoxifen (n = 8–9 mice). **r**, Frequencies of IL-17RB^+^ tuft cells in the indicated mice by flow cytometry (n = 3–5 mice). Data median (b,m-p) or mean ± s.e.m (e,f,i-l,q,r) is indicated. Data analyzed using Ordinary one-way ANOVA(b,r) or Mann-Whitney test (e,f,i-q). *P 0.01–0.05; **, P 0.01–0.001; ***, P 0.001–0.0001;****, P < 0.0001; ns, P ≥ 0.05.

Our proteomics data indicated distinct expression of IL-17RB in tuft cells (Fig. 2d), in line with previous findings by us and others that showed *Il17rb* mRNA expression in tuft cells compared to other intestinal epithelial cells of mice and humans^8,26^. Using flow cytometry, we further confirmed the expression of IL-17RB in tuft cells, which remained unchanged at both the protein and mRNA levels following succinate stimulation (Fig. 4a–c). Furthermore, we observed no activation of NF-κB transcriptional activity or substantial changes in the proteome of tuft cells exposed to IL-25, arguing against an autocrine signaling role (Extended Data Fig. 4a–c). This unusual coexpression of the ligand–receptor pair of IL-25 and IL-17RB prompted us to test the role of IL-17RB in the regulation of tuft cell IL-25. For this, we generated *Vil1*^Cre^; *Il17rb*^fl/fl^ mice, in which *Il17rb* is deleted in intestinal epithelial cells. The absence of IL-17RB staining was confirmed in tuft cells of *Vil1*^Cre^; *Il17rb*^fl/fl^ and *Il17rb*^–/–^ mice (Fig. 4a). To our surprise, tuft cell numbers were increased in the small intestine of *Vil1*^Cre^; *Il17rb*^fl/fl^ (Fig. 4d,e). The concomitant increase in goblet cells indicated an elevated basal activity of the tuft cell–ILC2 circuit in *Vil1*^Cre^; *Il17rb*^fl/fl^ mice (Extended Data Fig. 4d,e). Indeed, IL-13 expression and the total numbers of small intestinal ILC2s of *Vil1*^Cre^; *Il17rb*^fl/fl^ mice were significantly increased compared to ILC2s from their *Il17rb*^fl/fl^ littermates, whereas their Ki-67 expression did not noticeably differ (Fig. 4f–h and Extended Data Fig. 4f). ILC2s exhibited increased IL-17RB expression, with IL-13 expression predominantly localized to a subset of *Arg1*-YFP^–^IL-17RB^+^ ILC2s, a phenotype consistent with prior descriptions of IL-25-stimulated ILC2s in the small intestinal lamina propria^7,27^ (Extended Data Fig. 4g). RNA-sequencing analysis comparing ILC2s from *Vil1*^Cre^; *Il17rb*^fl/fl^ and *Il17rb*^fl/fl^ littermates revealed similar expression levels of ILC2 signature genes, whereas markers associated with ILC2 activation were upregulated in ILC2s from *Vil1*^Cre^; *Il17rb*^fl/fl^ mice (Fig. 4i). In contrast, lung ILC2s did not display activation, as indicated by the unchanged and low IL-13 and Ki-67 expression (Extended Data Fig. 4h–j). *Vil1*^Cre^; *Il17rb*^fl/fl^ mice exhibited a longer small intestine but maintained a normal body weight (Extended Data Fig. 4k,l). Additionally, *Vil1*^Cre^; *Il17rb*^fl/fl^ mice displayed efficient clearance of the helminth *N. brasiliensis* and a slight enhancement in resistance to *Heligmosomoides polygyrus* infection (Extended Data Fig. 4m–o). Collectively, these findings suggest a constitutive activation of the tuft cell–ILC2 circuit in *Vil1*^Cre^; *Il17rb*^fl/fl^ mice.

To investigate the dynamics of ILC2 activation following the deletion of tuft cell IL-17RB, we generated *Vil1*^CreERT2^; *Il17rb*^fl/fl^ mice and administered tamoxifen to adult mice. Consistent with our observations in *Vil1*^Cre^; *Il17rb*^fl/fl^ mice, ILC2s exhibited increased IL-13 expression at both 1 and 6 weeks following tamoxifen treatment (Fig. 4j). Moreover, Ki-67 expression in ILC2s was upregulated 1 week after tamoxifen treatment but returned to control levels by 6 weeks, when a concurrent increase in IL-17RB expression was also observed (Fig. 4k and Extended Data Fig. 4p). Notably, *Il25* mRNA expression was unchanged in tuft cells of *Vil1*^CreERT2^; *Il17rb*^fl/fl^ mice following *Il17rb* deletion (Extended Data Fig. 4q). To confirm that the elevated tonic activation of ILC2s in *Vil1*^CreERT2^ and *Vil1*^Cre^; *Il17rb*^fl/fl^ mice was a result of *Il17rb* deficiency in tuft cells, we generated *Il25*^iCre/+^; *Il17rb*^fl/fl^ animals, in which deletion is limited to tuft cells due to their unique expression of *Il25* (refs. ^3,28^). Although the recombination efficiency was lower than that in *Vil1*^Cre^; *Il17rb*^fl/fl^ mice, IL-17RB expression was depleted in most of the tuft cells of *Il25*^iCre/+^; *Il17rb*^fl/fl^ mice (Extended Data Fig. 4r). This resulted in ILC2s exhibiting elevated IL-13 expression and unchanged Ki-67 levels, along with a subtle increase in tuft cell abundance (Fig. 4l–n). These findings are consistent with the heightened activation of ILC2s observed in *Vil1*^CreERT2^ and *Vil1*^Cre^; *Il17rb*^fl/fl^ mice.

**Fig. 5:**
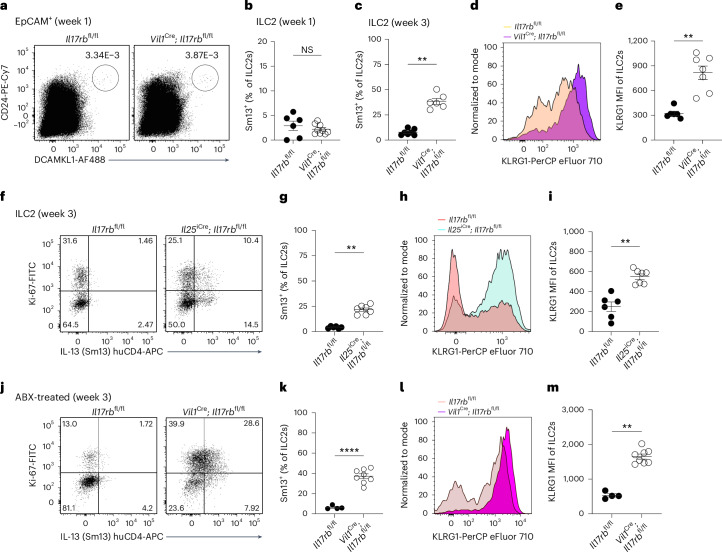
Tuft cell-intrinsic IL-17RB controls microbiota-independent ILC2 activation in young mice. **a**, Flow cytometry analysis of small intestinal tuft cells from *Vil1*^Cre^; *Il17rb*^fl/fl^ and *Il17rb*^fl/fl^ mice, gated as DCLK1^+^CD24^+^ cells of EpCAM^+^ cells. **b**,**c**, Frequencies of IL-13 (Sm13)^+^ ILC2s at 1 week (**b**) or 3 weeks (**c**) of age, quantified by flow cytometry (*n* = 6–9 mice). **d**,**e**, Expression (**d**) and quantification (**e**) of KLRG1 in ILC2s, analyzed by flow cytometry (*n* = 5–7 mice). **f**, Flow cytometry analysis of IL-13 (Sm13) reporter expression by ILC2s from 3-week-old *Il25*^iCre/+^; *Il17rb*^fl/fl^ and *Il17rb*^fl/fl^ mice. **g**, Quantification of IL-13 (Sm13)^+^ ILC2s (*n* = 6–7 mice). **h**,**i**, Flow cytometry analysis of KLRG1 expression by ILC2s (**h**) and quantification of the KLRG1 MFI (**i**) (*n* = 6–7 mice). **j**, Flow cytometry analysis of Ki-67 and Sm13 reporter expression by ILC2s from 3-week-old *Vil1*^Cre^; *Il17rb*^fl/fl^ and *Il17rb*^fl/fl^ mice, treated before and after birth with broad-spectrum antibiotics (ABX: metronidazole, neomycin sulfate, vancomycin and ampicillin) in drinking water. **k**, Quantification of IL-13 (Sm13)^+^ ILC2s (*n* = 4–8 mice). **l**,**m**, Flow cytometry analysis of KLRG1 expression by ILC2s (**l**) and quantification of the KLRG1 MFI (**m**) (*n* = 4–8 mice). Data were analyzed using the Mann–Whitney test and represented as mean ± s.e.m (**b**, **c**, **e**, **g**, **i**, **k**, **m**). **P = 0.01–0.001; ****P < 0.0001; NS, *P* ≥ 0.05.

IL-25^+^ tuft cells emerge in the small intestine shortly before weaning^7^. To test whether tuft cell IL-17RB controls ILC2 activity at an early age, we assessed IL-13 expression in ILC2s from 1- and 3-week-old mice. At 1 week, when tuft cells were scarce, IL-13 expression was low and did not differ between *Il17rb*^fl/fl^ and *Vil1*^Cre^; *Il17rb*^fl/fl^ mice (Fig. 5a,b). By 3 weeks, ILC2s from *Vil1*^Cre^; *Il17rb*^fl/fl^ mice exhibited elevated activation, with increased IL-13 and KLRG1 expression (Fig. 5c–e), findings confirmed in *Il25*^iCre/+^; *Il17rb*^fl/fl^ mice (Fig. 5f–i). As our animals were free of *Tritrichomonas* protists, a major driver of ‘homeostatic’ circuit activation^4,7^, we determined whether other intestinal microorganisms were responsible for ILC2 activation in mice with tuft cell IL-17RB deficiency. However, *Vil1*^Cre^; *Il17rb*^fl/fl^ offspring continued to show increased IL-13 and KLRG1 expression even after perinatal antibiotic treatment to deplete the microbiota (Fig. 5j–m). These results suggest that tuft cell-intrinsic IL-17RB regulates microbiota-independent ILC2 activation, a process that begins after birth as both cell types emerge in the small intestine and continues into adulthood.

### Tuft cell IL-17RB regulates tonic IL-25 availability to prevent excessive ILC2 activation

**Fig. 6:**
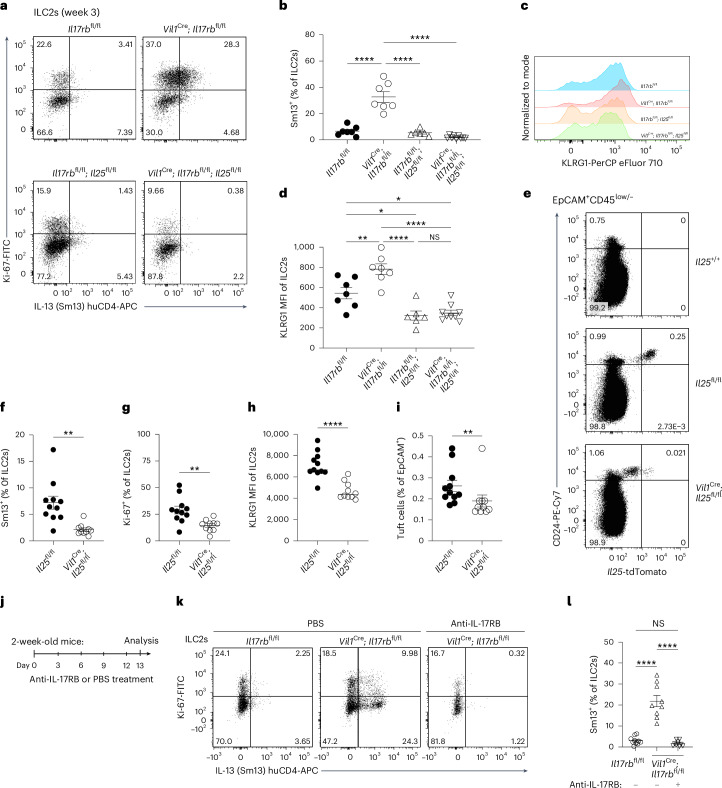
Tuft cell-intrinsic IL-17RB regulates tonic IL-25 bioavailability. **a**, Flow cytometry analysis of Ki-67 and Sm13 expression by small intestinal ILC2s from 3-week-old mice of the indicated genotypes. **b**, Quantification of IL-13 (Sm13)^+^ ILC2s (*n* = 6–9 mice). **c**, Analysis of KLRG1 expression by ILC2s. **d**, Quantification of KLRG1 MFI in ILC2s (*n* = 6–9 mice). **e**, Expression of tdTomato and CD24 in CD45^low/–^EpCAM^+^ cells determined by flow cytometry analysis from 3-week-old *Vil1*^Cre^; *Il25*^fl/fl^ and *Il25*^fl/fl^ mice, which also encode the *Il25*^tdTomato^ reporter, and compared to that in a reporter-negative mouse. **f**,**g**, Frequencies of IL-13 (Sm13)^+^ (**f**) and Ki-67^+^ (**g**) ILC2s (*n* = 10–11 mice). **h**, Quantification of the KLRG1 MFI in ILC2s (*n* = 10–11 mice). **i**, Quantification of tuft cells by flow cytometry (*n* = 10–11 mice). **j**, Experimental scheme. **k**, Flow cytometry analysis of Ki-67 and Sm13 reporter expression by ILC2s from young *Vil1*^Cre^; *Il17rb*^fl/fl^ and *Il17rb*^fl/fl^ mice after five injections of PBS or an anti-IL-17RB blocking antibody. **l**, Quantification of IL-13 (Sm13)^+^ ILC2s (*n* = 9–11 mice). Data are indicated as mean or mean ± s.e.m (**b**, **d**, **f**, **g**, **i**, **l**). Data were analyzed using an ordinary one-way ANOVA (**b**, **d**, **l**) or the Mann–Whitney test (**f**, **g**, **h**, **i**). *P = 0.01–0.05; **P = 0.01–0.001; ****P < 0.0001; NS, *P* ≥ 0.05.

**Extended Data Fig. 5:**
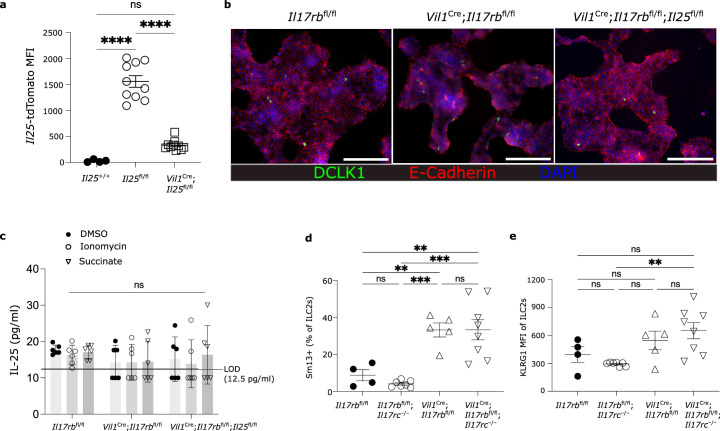
Elevated tonic ILC2 stimulation in *Vil1*^Cre^;*Il17rb*^fl/fl^ mice is independent of *Il17rc.* **a**, *Il25*^tdTomato^ MFI of EpCAM^+^ CD24^+^SiglecF^+^ tuft cells were determined by flow cytometry from 3-week-old *Vil1*^Cre^;*Il25*^fl/fl^ and *Il25*^fl/fl^ mice which also encode the *Il25*^tdTomato^ reporter, and compared to that in a reporter-negative mouse (n = 4–10 mice). **b**, Representative immunofluorescence image of DCLK1 (green) in 2D organoids from mice of the indicated genotypes (scale bars, 200 μm). **c**,IL-25 production in supernatants of 2D organoids stimulated with DMSO, 1 µg/mL ionomycin, or 50 mM dibasic sodium succinate hexahydrate for 30 min. The dashed line indicates the limit of detection (n = 6 samples from 6 mice). **d**,**e**, Enumeration of IL-13 (Sm13)^+^ ILC2 percentage (d) and quantification of the KLRG1 MFI in ILC2s (e) (n = 4–8 mice). Data are represented as mean ± s.e.m (a,c-e). Data was analyzed using Ordinary one-way ANOVA (a,c-e). **, P 0.01–0.001; ***, P 0.001–0.0001;****, P < 0.0001; ns, P ≥ 0.05.

As tuft cells constitutively express the *Il25* transcript, we hypothesized that deleting the cognate receptor IL-17RB in tuft cells would lead to increased IL-25 release and, consequently, the activation of ILC2s. To test whether IL-25 is required for the heightened basal activation state of ILC2s in mice lacking IL-17RB in tuft cells, we generated *Vil1*^Cre^; *Il17rb*^fl/fl^; *Il25*^fl/fl^ mice. Indeed, the absence of both IL-17RB and IL-25 abrogated the elevated expression of IL-13 and KLRG1 observed in 3-week-old mice (Fig. 6a–d). These results suggest a constant low-level production of IL-25, which is sensed by ILC2s. In agreement with prior data, we found that tuft cells also readily express the *Il25*^tdTomato^ reporter in young mice (Fig. 6e)^7^. To assess the consequence of IL-25 deficiency on the basal activation state of ILC2s, we analyzed *Vil1*^Cre^; *Il25*^fl/fl^ mice. Reduced tdTomato expression in tuft cells of *Vil1*^Cre^; *Il25*^fl/fl^ mice confirmed efficient Cre-mediated *Il25* deletion (Fig. 6e and Extended Data Fig. 5a). Compared to their littermate controls, ILC2s from *Vil1*^Cre^; *Il25*^fl/fl^ mice showed significantly lower levels of IL-13, Ki-67 and KLRG1 expression (Fig. 6f–h), consistent with our observations in mice with ILC2-intrinsic *Il17rb* deletion (Fig. 1a–d and Extended Data Fig. 1e). Furthermore, the frequency of tuft cells among epithelial cells was reduced in the absence of IL-25 (Fig. 6i). Overall, this suggests that tuft cells constitutively produce low levels of IL-25, which maintains basal ILC2 activation.

Despite the presence of tuft cells, we did not detect IL-25 protein expression above the background level in stimulated two-dimensional (2D) organoids, in agreement with prior studies (Extended Data Fig. 5b,c)^6^. To demonstrate further that the tonic tuft cell IL-25–ILC2 axis drives excessive ILC2 activation when quenching by tuft cell IL-17RB is lacking, we treated 2-week-old *Vil1*^Cre^; *Il17rb*^fl/fl^ mice with an anti-IL-17RB blocking antibody, enabling the inhibition of IL-17RB engagement by IL-25 protein in ILC2s with temporal control (Fig. 6j). This treatment abolished the features of increased basal ILC2 activation in *Vil1*^Cre^; *Il17rb*^fl/fl^ mice (Fig. 6k,l). As IL-17RB forms a receptor complex with the shared receptor component IL-17RA, we considered that the absence of IL-17RB may result in altered receptor chain pairing of IL-17RA, potentially enhancing signaling through the IL-17RA–IL-17RC complex, which has recently been suggested to promote secretory cell differentiation in the small intestine^29^. To rule out this possibility, we crossed *Vil1*^Cre^; *Il17rb*^fl/fl^ mice with *Il17rc*^–/–^ mice. These *Vil1*^Cre^; *Il17rb*^fl/fl^; *Il17rc*^–/–^ mice still exhibited increased IL-13 expression, similar to *Vil1*^Cre^; *Il17rb*^fl/fl^ mice, demonstrating that IL-17A and IL-17F signaling is not involved (Extended Data Fig. 5d,e). We conclude that tuft cells constitutively express the *Il25* transcript and produce small amounts of IL-25 protein, which contributes to basal ILC2 activation in the small intestine. Tuft cell IL-17RB expression regulates bioavailable IL-25 levels in the lamina propria, thereby modulating homeostatic tuft cell–ILC2 circuit activity.

### Prolonged activation by IL-25 induces a hypoproliferative state in ILC2s

**Fig. 7:**
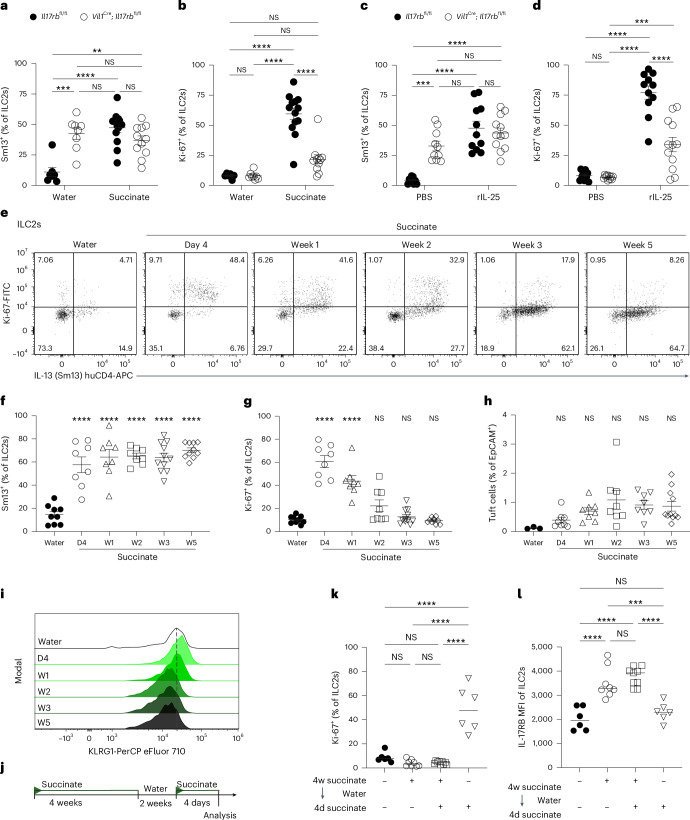
Prolonged activation by IL-25 induces a hypoproliferative state in ILC2s. **a**,**b**, Percentage of IL-13 (Sm13)^+^ (**a**) and Ki-67^+^ (**b**) ILC2s in the small intestine quantified by flow cytometry from adult *Vil1*^Cre^; *Il17rb*^fl/fl^ and *Il17rb*^fl/fl^ littermate mice treated with succinate for 4 days (*n* = 6–13 mice). **c**,**d**, Percentage of IL-13 (Sm13)^+^ (**c**) and Ki-67^+^ (**d**) ILC2s in the small intestine quantified by flow cytometry from adult *Vil1*^Cre^; *Il17rb*^fl/fl^ and *Il17rb*^fl/fl^ littermate mice treated with 1 μg rIL-25 on two consecutive days (*n* = 9–12 mice). **e**, Flow cytometry analysis of Ki-67 and IL-13 (Sm13) reporter expression by ILC2s from IL-13 (Sm13) reporter mice treated with succinate for the indicated amount of time. **f**,**g**, Frequencies of IL-13 (Sm13)^+^ (**f**) and Ki-67^+^ (**g**) ILC2s (*n* = 8–10 mice; D, days; W, weeks). **h**, Percentage of DCLK1^+^CD24^+^ tuft cells among epithelial cells (*n* = 8–10 mice). **i**, Representative histograms displaying KLRG1 expression by ILC2s. **j**, Mice were treated with succinate for 4 weeks, followed by 2 weeks of regular drinking water and another 4 days of succinate treatment, and the small intestine was analyzed. **k**,**l**, Frequencies of Ki-67^+^ ILC2s (**k**) and MFI of IL-17RB in ILC2s (**l**) analyzed by flow cytometry (*n* = 6–9 mice). Data are indicated as mean ± s.e.m (**a**–**d**, **f**–**h**) or median (**k**, **l**). Data were analyzed using a two-way ANOVA (**a**–**d**) or an ordinary one-way ANOVA (**k**, **l**). **P = 0.01–0.001; ***P = 0.001–0.0001; ****P < 0.0001; NS, *P* ≥ 0.05.

To understand better the functional consequences of uncontrolled tonic IL-25 release and the resulting prolonged stimulation of ILC2s, we treated adult *Vil1*^Cre^; *Il17rb*^fl/fl^ mice with succinate and assessed ILC2 activation 4 days later. IL-13 expression was comparable between ILC2s from *Vil1*^Cre^; *Il17rb*^fl/fl^ and littermate control mice after tuft cell stimulation with succinate, matching the elevated expression already observed in ILC2s from *Vil1*^Cre^; *Il17rb*^fl/fl^ mice at baseline (Fig. 7a). However, while both groups exhibited minimal baseline Ki-67 expression without treatment, ILC2s in *Vil1*^Cre^; *Il17rb*^fl/fl^ mice failed to upregulate Ki-67 upon succinate administration, unlike ILC2s in their littermate controls, which showed robust proliferative capacity (Fig. 7b). As succinate induces IL-25 release from tuft cells, we then bypassed tuft cells by directly injecting rIL-25 into *Vil1*^Cre^; *Il17rb*^fl/fl^ and *Il17rb*^fl/fl^ control mice. Consistent with the succinate results, ILC2s from both genotypes robustly expressed IL-13, whereas the proliferative response, indicated by Ki-67 expression, was reduced in ILC2s from *Vil1*^Cre^; *Il17rb*^fl/fl^ mice (Fig. 7c,d). Therefore, we conclude that ILC2s in *Vil1*^*Cre*^; *Il17rb*^fl/fl^ mice, chronically exposed to elevated tonic IL-25, had become hyporesponsive toward additional IL-25 as elicited by tuft cell stimulation.

To test this hypothesis in a nongenetic model, we treated wild-type mice—carrying only encoded reporter alleles—with succinate over varying times to sustain tuft cell IL-25-mediated ILC2 activation (Fig. 7e). Regardless of the duration of stimulation, IL-13 expression in ILC2s remained elevated up to 5 weeks, consistent with ongoing tuft cell stimulation by succinate (Fig. 7e,f). In contrast, the percentage of Ki-67^+^ ILC2s steadily declined following the initial peak, returning to levels similar to those in untreated mice after 5 weeks (Fig. 7e,g). This decline in Ki-67 expression occurred despite the modest increase in tuft cell frequencies and was accompanied by a gradual reduction in ILC2 KLRG1 expression (Fig. 7h,i), mirroring the findings from *Vil1*^Cre^; *Il17rb*^fl/fl^ mice exposed to chronically elevated tonic IL-25 caused by tuft cell *Il17rb* deficiency.

**Extended Data Fig. 6:**
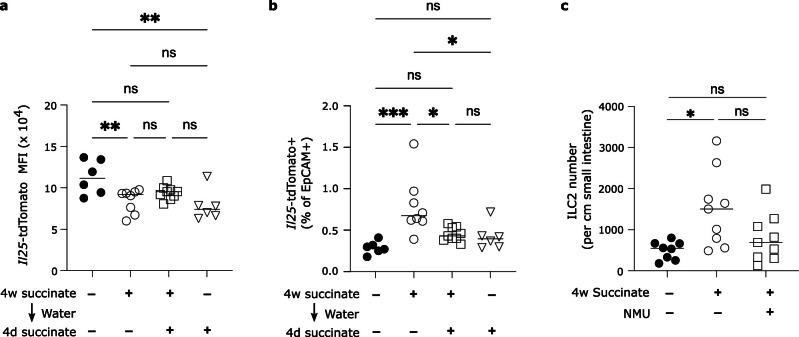
Prolonged activation by IL-25 induces a hypoproliferative state in ILC2s. **a**,**b**, The MFI of *Il25*^tdTomato^ reporter in tuft cells (a) and the percentages of *Il25*^tdTomato+^ cells (b) were quantified by flow cytometry. The SI was analyzed from mice treated with succinate for 4 weeks, followed by 2 weeks of regular drinking water, and another 4 days of succinate treatment, according to the schematic in (Fig. 7j), (n = 6–9 mice). **c**, The number of SI ILC2s was quantified from IL-13 (Sm13)-reporter mice treated with succinate for 4 weeks, followed by neuromedin U (NMU) injections on two consecutive days. (n = 8–9 mice). Data are represented as median (a-c). Data were analyzed using Ordinary one-way ANOVA(a-c). *, P 0.01–0.05; **, P 0.01–0.001; ***, P 0.001–0.0001; ns, P ≥ 0.05.

Notably, the hypoproliferative state of ILC2s did not immediately revert. We treated wild-type mice with succinate for 4 weeks, allowed them to rest on regular drinking water for 2 weeks and reexposed them to succinate for 4 days (Fig. 7j). Despite comparable *Il25* expression in tuft cells and a slightly elevated tuft cell percentage, ILC2 Ki-67 expression remained significantly lower in the pretreated group, whereas IL-17RB expression increased (Fig. 7k,l and Extended Data Fig. 6a,b). Furthermore, the 2-week duration is long enough for the generation of new tuft cells that had not been previously exposed to succinate, making it unlikely that their response to the agonist would be atypical. Therefore, we conclude that prior IL-25 stimulation is most likely responsible for inducing a state in resident ILC2s that hinders their sustained proliferative capacity.

### A hypoproliferative ILC2 state associated with chronic helminth infection

**Fig. 8:**
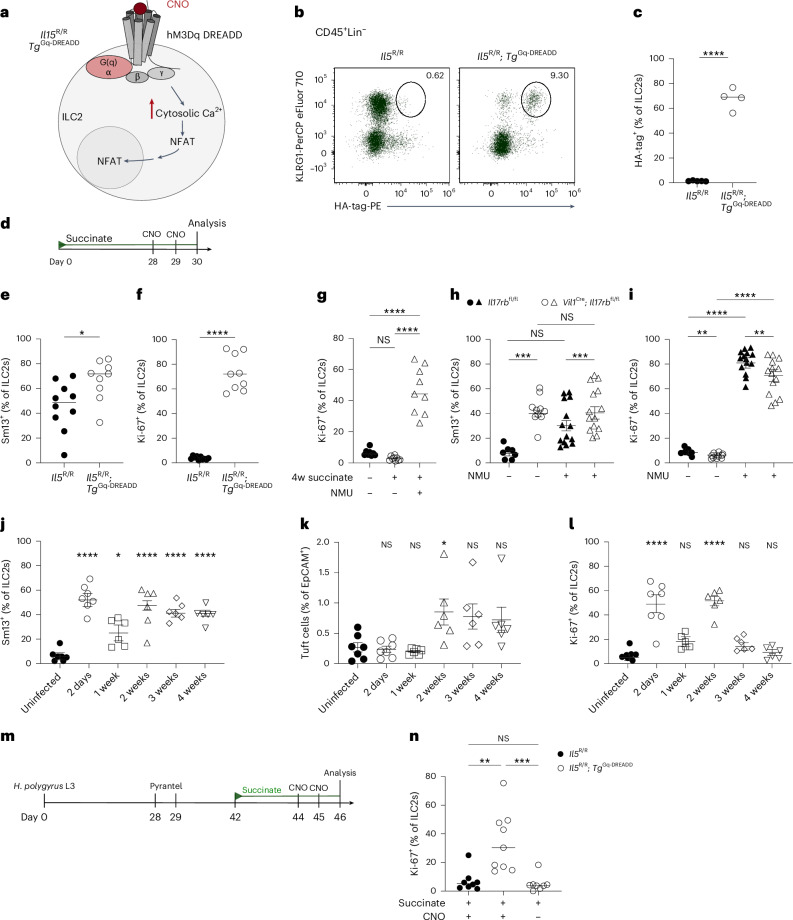
Stimulation of a nonredundant signaling pathway restores proliferative capacity in ILC2s. **a**, Schematic of the signaling pathway engaged by the hM3Dq DREADD agonist CNO. **b**, KLRG1 and hM3Dq DREADD (HA-tag) in ILC2s, gated on CD45^+^Lin^–^ cells from *Il5*^R/R^ and *Il5*^R/R^; *Tg*^Gq-DREADD^ mice. **c**, Quantified percentage of hM3Dq DREADD^+^ ILC2s (*n* = 4–5 mice). **d**, Schematic showing 30-day succinate treatment with CNO injections on two consecutive days before analysis; used in **e** and **f**. **e**,**f**, Frequencies of small intestinal IL-13 (Sm13)^+^ (**e**) and Ki-67^+^ (**f**) ILC2s from *Il5*^R/R^ and *Il5*^R/R^; *Tg*^Gq-DREADD^ mice quantified by flow cytometry (*n* = 9–10 mice). **g**, IL-13 (Sm13) reporter mice were treated with succinate for 4 weeks, followed by NMU injections on two consecutive days. The frequencies of Ki-67^+^ ILC2s were analyzed by flow cytometry (*n* = 8–9 mice). **h**,**i**, Percentages of IL-13 (Sm13)^+^ (**h**) and Ki-67^+^ (**i**) small intestinal ILC2s analyzed by flow cytometry from *Vil1*^Cre^; *Il17rb*^fl/fl^ and *Il17rb*^fl/fl^ mice treated with NMU for two consecutive days (*n* = 7–14 mice). **j**–**l**, YRS reporter mice infected with *H. polygyrus* were analyzed at the indicated time points. The frequencies of small intestinal IL-13 (Sm13)^+^ ILC2s (**j**), quantification of tuft cells (**k**) and frequencies of Ki-67^+^ ILC2s (**l**) were analyzed by flow cytometry (*n* = 6–7 mice). **m**, *Il5*^R/R^ and *Il5*^R/R^; *Tg*^Gq-DREADD^ mice were infected with third-stage larvae (L3) of *H. polygyrus*. Worms were cleared with pyrantel pamoate. Two weeks later, mice were treated with succinate water for 4 days and injected with CNO on two consecutive days before analysis. Control groups consisted of *Il5*^R/R^ mice also treated with CNO and *Il5*^R/R^; *Tg*^Gq-DREADD^ mice treated with PBS. **n**, Frequencies of Ki-67^+^ ILC2s in the small intestinal lamina propria analyzed by flow cytometry (*n* = 8–9 mice). Data are indicated as mean (**c**–**g**, **n**) or mean ± s.e.m (**h**–**i**). Data were analyzed using a two-tailed unpaired *t* test (**c**, **e**, **f**) or an ordinary one-way ANOVA (**g**–**l**, **n**). *P = 0.01–0.05; **P = 0.01–0.001; ***P = 0.001–0.0001; ****P < 0.0001; NS, *P* ≥ 0.05.

ILC2s express receptors that allow them to integrate diverse molecular signals from their surrounding tissue environment^30^. We speculated that the stimulation of tuft cells with an agonist such as succinate, specifically triggering the release of IL-25 but no robust production of other effector molecules that activate ILC2s, might be insufficient to enable proliferation over multiple cycles. To test this hypothesis, we thought to provide stimulation with an additional signaling modality in a highly specific manner. To that end, we crossed YRS mice, expressing Cre in ILC2s, with a *Tg*^CAG-LSL-Gq-DREADD^ strain. This generated *Il5*^R/R^; *Tg*^Gq-DREADD^ mice in which ILC2s overexpress a mutant hM3Dq GPCR that induces the canonical Gq pathway specifically following administration of the molecule clozapine-*N*-oxide (CNO) (Fig. 8a–c). This model enabled us to mimic GPCR–Gq–Ca^2+^–NFAT (nuclear factor of activated T cells) signaling as induced downstream of receptor activation by leukotrienes or neuromedin U (NMU), known to contribute to ILC2 activation^30^. *Il5*^R/R^; *Tg*^Gq-DREADD^ and *Il5*^R/R^ littermate control mice were then treated with succinate for 4 weeks to induce a hypoproliferative state, and CNO was injected twice over the last 2 days (Fig. 8d). In agreement with our prior results, control mice without Gq-DREADD overexpression showed substantial IL-13 expression but lacked signs of proliferation as assessed by Ki-67 staining (Fig. 8e,f). In contrast, most ILC2s from *Il5*^R/R^; *Tg*^Gq-DREADD^ mice became Ki-67^+^ upon CNO treatment (Fig. 8f). Similarly, injecting NMU into mice after 4 weeks of succinate exposure produced results comparable to those observed with CNO injection (Fig. 8g). These results suggest that another, nonredundant, pathway together with IL-25 signaling is necessary to maintain proliferative capacity.

To test whether Ki-67 expression could, in a similar manner, also be restored in ILC2s from *Vil1*^Cre^; *Il17rb*^fl/fl^ mice, we treated adult animals with NMU at a time point when their response to succinate or IL-25 was significantly impaired (Fig. 7b,d). NMU injection induced the expression of IL-13 and Ki-67 in ILC2s from control mice, in accordance with prior studies (Fig. 8h,i)^31–33^. While NMU did not further increase the already elevated baseline IL-13 expression in *Vil1*^Cre^; *Il17rb*^fl/fl^ mice, it significantly increased the frequency of Ki-67^+^ ILC2s, which were previously unable to proliferate when exposed to IL-25 or the IL-25-inducing stimulus succinate (Fig. 7b,d). Together, these results demonstrate that the combined action of at least two nonredundant pathways is necessary to ensure full proliferative capacity in ILC2s.

Chronic helminth infection is marked by an elevation in tuft cell numbers, although this response alone has been shown ineffective in eradicating adapted parasites such as *H. polygyrus*^3,4,34^. We hypothesized that a hypoproliferative state might also be induced in ILC2s under these conditions of persistent tuft cell–ILC2 stimulation, further contributing to the diminished type 2 responses, in addition to the direct immunomodulatory tactics used by this parasite^35^. To assess this, we analyzed features of tuft cell–ILC2 circuit activation in YRS mice at different time points following infection with *H*. *polygyrus*. IL-13 expression by ILC2s was detected shortly after infection and persisted throughout the entire period when adult worms were present in the lumen, extending up to 4 weeks (Fig. 8j). The frequency of IL-13^+^ ILC2s decreased 1 week after infection, when worm larvae migrated away from the intestinal lumen and temporarily took up residence close to the outer muscularis layer, where they might be shielded from tuft cell detection (Fig. 8j). In parallel, tuft cell frequencies increased as reported previously (Fig. 8k). Notably, the percentages of Ki-67^+^ ILC2s mirrored the IL-13 expression pattern, characterized by an initial increase, a temporary decline and a resurgence when adult worms colonized the lumen of the small intestine (Fig. 8l). However, the frequency of Ki-67^+^ ILC2s gradually decreased over the following 2 weeks, eventually reaching a level akin to that of uninfected mice (Fig. 8l).

To test whether ILC2s indeed acquired a lasting hypoproliferative state, we cleared the worms after 4 weeks and rested the mice for an additional 2 weeks before treatment with succinate to stimulate IL-25-mediated ILC2 proliferation (Fig. 8m). By performing these experiments in *Il5*^R/R^ and *Il5*^R/R^; *Tg*^Gq-DREADD^ mice, we were also able to test directly the consequences of stimulating the Gq-DREADD pathway with CNO. Consistent with our prior observations, ILC2s from control groups failed to upregulate Ki-67, indicating an impaired proliferative response when stimulated through the succinate–IL-25 axis following helminth clearance (Fig. 8n). In contrast, injection of CNO during succinate stimulation significantly increased the frequency of Ki-67^+^ ILC2s in *Il5*^R/R^; *Tg*^Gq-DREADD^ mice (Fig. 8n). Overall, these results identify a hypoproliferative state in ILC2s following prolonged circuit activation due to chronic helminth infection, which could be overcome by providing stimulation through a separate, nonredundant signaling pathway.

## Discussion

The investigation into the cytokine IL-25 has had a pivotal role in uncovering the existence of ILC2s and subsequently elucidating the involvement of tuft cells in initiating innate type 2 responses within the small intestine. Our research has yielded several crucial insights into the regulation of this cytokine in the predominant cell types orchestrating these responses. First, we identified key mechanistic steps governing the production or secretion of IL-25 by tuft cells, triggered by the detection of succinate before subsequent activation of ILC2s. Second, building on the implications from previous studies on IL-25 and ILC2s, we unequivocally demonstrated the ILC2-intrinsic requirement for IL-17RB during physiological stimulation of the tuft cell–ILC2 circuit. Third, we uncovered an unsuspected regulatory function of IL-17RB in tuft cells, controlling the levels of bioavailable IL-25 and thereby preventing excessive tonic stimulation of ILC2s resulting from constitutive expression of the *Il25* transcript in tuft cells. Lastly, we described a hypoproliferative state in ILC2s induced by chronic IL-25 exposure, which could be offset by the engagement of nonredundant pathways such as NFAT activation.

The discovery that tuft cells can respond to succinate and subsequently stimulate small intestinal ILC2s in an IL-25-dependent manner has provided the field with a straightforward and physiologically relevant model for studying the tuft cell–ILC2 circuit^7–9^. Tuft cells constitutively express *Il25* mRNA in vivo and in vitro, even in the absence of agonistic molecules such as succinate^3^. In agreement with prior studies, possibly influenced by the presence of *Tritrichomonas* protists, we could confirm constitutive *Il25* transcript expression in SOPF mice. Tuft cells from neonatal mice free of *Tritrichomonas* expressed *Il25* as soon as they emerged in the small intestinal epithelium, consistent with previous reports^7^. Constitutive expression of the *Il25* transcript might reflect the anticipatory state in which tuft cells are engaged, also marked by concurrent expression of the enzymatic machinery for the production of leukotrienes and acetylcholine^6,8,36,37^. The constant de novo differentiation of tuft cells from the pool of leucine-rich repeat-containing GPCR 5 (LGR5)^+^ stem cells, coupled with their short life span in the villus epithelium, likely requires such a state of preparedness to facilitate immediate and meaningful responses to luminal signals^28,38^. Indeed, we found no evidence of *Il25* mRNA upregulation when tuft cells were stimulated with succinate. However, IL-25 protein becomes rapidly available to activate ILC2s, which we could prevent using an antibody that blocks the engagement of the receptor IL-17RB by IL-25.

The post-transcriptional regulation of IL-25 protein involves a series of intracellular events that are just beginning to be unraveled. Prior studies demonstrated the need for tuft cell phospholipase Cβ2 (PLCβ2) and TRPM5—both highly expressed in tuft cells—in succinate-elicited, tuft cell-mediated activation of ILC2s and the associated expansion of tuft cells^7–9,39^. Our work highlights the critical role of Ca^2+^ release from intracellular stores in the endoplasmic reticulum, mediated by IP3R2, which we demonstrated using *Itpr2*^–/–^ mice and live cell calcium imaging of tuft cells in intact villi. The control of IL-25 translation and/or its release by intracellular Ca^2+^ elevation, possibly involving Ca^2+^-activated TRPM5-mediated depolarization, merits further investigation. Thus, unlike taste cells in the oral taste bud, which signal in an IP3R3-dependent manner, small intestinal tuft cells use IP3R2 (ref. ^24^). High expression of *Itpr2* in tuft cells from the human intestine indicates that this function might be conserved^26,40^. In tracheal tuft cells, an elevation in intracellular calcium is linked to acetylcholine release that stimulates surrounding epithelial cells and triggers Ca^2+^ waves, thereby promoting ciliary activity and Cl^−^ secretion^22^. Small intestinal tuft cells may use similar mechanisms to produce IL-25. Whether small intestinal tuft cells also depend on IP3R2 for TRPM5-mediated acetylcholine release and fluid secretion, or for helminth-induced leukotriene mobilization, requires further study^36,37^. A recent report showing impaired helminth-evoked type 2 responses in mice lacking lymphoid-restricted membrane protein (LRMP), an endoplasmic reticulum-resident protein and possible interaction partner of IP3Rs, suggests that IP3R2 activation may indeed be a central signaling step downstream of multiple tuft cell stimuli^41^.

Numerous reports have underscored the interdependence between tuft cells, IL-25 and ILC2s in facilitating type 2 responses, as observed in helminth infections, or during heightened luminal succinate levels^3,4,7–9,34^. Early studies showed that IL-25 can also activate ILC2s when isolated and stimulated in vitro^17,18^. The high expression of IL-17RB in small intestinal ILC2s, coupled with numerous subsequent investigations using IL-25 as a potent ILC2 agonist, has established the model whereby ILC2s directly respond to IL-25, known as the tuft cell–ILC2 circuit^42^. However, this assumption has not been formally verified under conditions of physiological stimulation of the circuit in vivo. In this study, we used genetic tools enabling the specific deletion of IL-17RB in ILC2s. Our findings unequivocally demonstrate an intrinsic requirement for IL-17RB in ILC2s to foster proliferation and induce IL-13 expression upon succinate-mediated circuit stimulation, as well as to mount effective responses to clear helminth infections. Collectively, the presented data solidify ILC2s as a direct target of tuft cell-derived IL-25.

The presence of the *Il17rb* transcript as an unexpected feature of tuft cells was noted before the identification of their role as drivers of type 2 immunity and was later confirmed by transcriptional profiling of tuft cells in mice and humans^8,26,40,43,44^. Our results provide further evidence for an anticipatory state of tuft cells wherein they sustain *Il25* transcripts in the absence of luminal triggers yet in which intrinsic IL-17RB expression regulates the tonic levels of bioavailable IL-25. The feed-forward nature of the circuit likely requires tight control to enable a rapid response while still preventing inappropriate steady-state stimulation, which is further supported by mechanisms constraining activation in ILC2s, such as A20 and CISH^7,19^. This threshold can be overcome by tuft cell agonists, such as succinate, that trigger swift IL-25-mediated circuit activation. Whether agonistic signaling lowers the activity of the regulatory breaks or simply stimulates the production of IL-25 protein to levels exceeding the quenching activities requires further investigation. Our study also did not address whether tuft cell IL-17RB only sequesters IL-25 or whether this interaction governs additional regulatory signaling in tuft cells. Recent studies linking IL-25 and IL-17RB to intestinal cancer warrant further characterization of this cytokine axis^45–47^.

Intriguingly, we describe a hypoproliferative state in ILC2s resulting from continuous stimulation by IL-25, induced either by genetic deletion of IL-17RB in tuft cells or by prolonged succinate exposure. This decline in ILC2 proliferation may reflect natural adaptation to states of prolonged tuft cell stimulation, providing an additional layer of control to prevent excessive circuit activity. Indeed, continuous low-grade stimulation of tuft cells by succinate or possibly other microbial agonists may be more common in a natural environment with dynamic alterations in the luminal state compared to the stable and low type 2 tone in mice housed in barrier facilities. Such circuit activation might be sufficient to provoke responses mediating adaptive alteration in antimicrobial programs^48,49^, yet it may not promote the strong expansion of tuft and goblet cells seen with *N. brasiliensis* infection^3,4,34^. We hypothesize that the ILC2 response is controlled by the cumulative effect of activating signaling pathways, which are integrated early during activation to subsequently determine the magnitude of proliferation, akin to the model proposed for T cells^50^. Prolonged supraoptimal stimulation by IL-25 alone resulted in impaired proliferation upon subsequent acute succinate challenge, suggesting that ILC2s retain a memory of prior stimulation. This phenomenon may involve epigenetic or other ILC2-intrinsic alterations and possibly extrinsic control of the ILC2 pool size based on available niche signals, thus reflecting adaptation to recurrent colonization or transient bursts of succinate-producing microorganisms that do not necessitate strong ILC2 expansion. However, further mechanistic studies are needed to explore this issue. Our findings parallel the ‘law of initial value’ by Wilder^51^, which describes how the basal state of activity can influence the response to subsequent stimuli, a concept recently revisited in the context of T cell responses^52^. Notably, although prior studies reported states of ILC2 exhaustion, we show that engaging synergistic NFAT signaling pathways can rapidly reverse the hypoproliferative state when optimal amounts of ILC2 stimulation are available^53,54^.

A consequence of ILC2s transitioning to a hypoproliferative state may be the impaired clearance of adapted helminths such as *H. polygyrus*. Under these circumstances, imbalanced levels of ILC2 stimuli may persist despite the continued abundance of luminal worms. Consequently, the induction of hypoproliferation could represent an additional manipulation in host cell responsiveness, akin to the recently described shaping of the epithelial response to IL-13 (ref. ^55^). Notably, the antagonism of IL-33 activity may contribute to the suboptimal level of ILC2 stimulation, facilitating the hypoproliferative state. Further studies are needed to dissect the alterations induced by chronic helminth infections in the activity of individual pathways acting on ILC2s, including those involving epithelial cytokines, eicosanoids and neuropeptides^30^. These mechanisms may unveil the elegant immunomodulatory potential of helminths, beyond the direct antagonistic action of secreted parasite products^35^. Overall, such adaptive responses in the host may be beneficial if productive clearance cannot be achieved, and excessive chronic type 2 stimulation might lead to undesirable effects.

## Methods

### Animal studies and mouse models

We used the following mouse strains: *Vil1*^Cre^ (B6.Cg-Tg(*Vil1*-*cre*)997Gum/J, The Jackson Laboratory (JAX), 004586); *Vil1*^CreERT2^, *Nmur1*^iCre-eGFP^ (refs. ^20,21^; from D. Artis and C. Klose); *Il17rb*^fl/fl^ (ref. ^15^; from U. Siebenlist); *Il17rb*^–/–^ mice with global *Il17rb* deletion, generated from *Vil1*^Cre^; *Il17rb*^fl/fl^ mice in which Cre is active with high frequency in the male germline; *Itpr2*^–/–^ (ref. ^56^; from A. Saab); *Trpm5*^Cre^; *R26*^GCaMP6f^ (refs. ^22,23^); *Il17rc*^–/–^ (Amgen, from S. LeibundGut-Landmann); *Il5*^Red5^ (B6(C)-*Il5*^*tm1.1(iCre)Lky*^/J, JAX, 030926); *Il25*^fl-tdTomato^ (ref. ^3^) (B6(C)-*Il25*^*tm1.1Lky*^/J); *Il25*^iCre^ (ref. ^28^) (B6(C)-*Il25*^*tm2.1(cre)Lky*^/J); *Tg*^CAG-LSL-Gq-DREADD^ (B6N;129-Tg(CAG-CHRM3*,-mCitrine)1Ute/J, JAX, 026220); B6(C)-*Gt(ROSA)26Sor*^*tm1(Nfkb-destEGFP)Kopf*^ (ref. ^57^). Except for *Trpm5*^Cre^; *R26*^GcaMP6f^ and NF-κB reporter mice, all lines were crossed with *Arg1*^Yarg^; *Il13*^Sm13^ double-reporter mice (B6.129S4-*Arg1*^*tm1Lky*^/J, JAX, 015857; B6.129S4(C)-*Il13*^*tm2.1Lky*^/J, JAX, 031367). *Arg1*^Yarg^; *Il5*^Red5^; *Il13*^Sm13^ triple-reporter mice are termed ‘YRS’. All mice were on a C57BL/6 background. Mice were bred and housed at the University of Zurich, Laboratory Animal Sciences Center in Zurich, Switzerland, under specific pathogen-free conditions, free of *Tritrichomonas*. Animals were housed in individually ventilated cage units containing autoclaved bedding and nesting material, with a 12-h light–dark cycle, under controlled temperature (18–23 °C) and humidity (40–60%), with ad libitum standard diet and water. Animal experiments were reviewed and approved by the cantonal veterinary office of Zurich (permit numbers 054/2022 and 111/2022) and in accordance with the guidelines established by the German Animal Welfare Act, European Communities Council Directive 2010/63/EU and the institutional ethical and animal welfare guidelines of Saarland University (approval number of the Institutional Animal Care and Use Committee: CIPMM-2.2.4.1.1). Age-matched mice of both sexes were used at age 6 weeks or older, except for the indicated experiments with pups. Experiments were performed with mice randomly allocated to groups and without investigator blinding. No statistical methods were used to predetermine sample sizes; sample sizes were similar to those reported in previous publications. No data points or animals were excluded from the analyses for any reason other than an animal becoming sick or injured or dying of causes unrelated to the experiment.

### Mouse treatments

Succinate treatment: 100 mM succinic acid (S3674, Sigma) was dissolved in autoclaved water, pH-neutralized with NaOH and filtered (0.22 μm). Mice were treated for the indicated duration with succinate instead of regular drinking water, and the solution was exchanged weekly. Anti-IL-17RB treatment: a monoclonal blocking antibody against IL-17RB (clone D9.2)^18^ was purified in-house and administered intraperitoneally at the indicated time points (antibody injection dose: adult mice, 200 μg; pups, 50 μg). Antibiotic treatment: breeders received drinking water supplemented with ampicillin (1 mg ml^−1^), metronidazole (1 mg ml^−1^), neomycin (1 mg ml^−1^), vancomycin (0.5 mg ml^−1^) and sucrose (1%) starting approximately 3 weeks before the birth of a litter until the offspring reached 3 weeks of age. IL-25 treatment: adult mice received 1 μg mouse rIL-25–hFc fusion protein (purified in-house) intraperitoneally on two consecutive days before killing. For proteomics analysis, mice were treated one time with 1.5 μg mouse rIL-25–hFc, 16 h before killing. CNO treatment: adult mice received intraperitoneal injections of water-soluble CNO dihydrochloride, 20 μg in 200 μl, two consecutive days before killing. NMU treatment: adult mice received intraperitoneal injections of mouse NMU-23 peptide (H-FKAEYQSPSVGQSKGYFLFRPRN-NH2, Mimotopes), 20 μg in 200 μl, two consecutive days before killing. Helminth infections: adult mice were infected subcutaneously with 500 infectious third-stage larvae of *N. brasiliensis* or by oral gavage with 200 infectious third-stage larvae of *H. polygyrus*. For worm clearance, mice were treated on two consecutive days by oral gavage with 2 mg pyrantel pamoate (Perrigo, 36200).

### Gut length

Immediately after the mice were killed, the small intestine was cut distal to the stomach, carefully pulled out of the abdomen and detached from mesenteric tissue. After cutting proximal to the cecum, the length of the small intestine was measured by hanging it vertically alongside a measuring tape.

### Single-cell tissue processing

Adult mice were killed by carbon dioxide, and pups were killed by decapitation. For the small intestine samples, as previously described^58^, single-cell suspensions were prepared using either the ‘basic protocol’ or ‘alternate protocol 1’ depending on the expected inflammatory status (that is, resting mice = basic protocol, following type 2 stimulation = alternate protocol). It was important for successful staining of IL-17RB that the basic protocol was used. In short, tissue from the proximal small intestine of adult mice or the complete intestine from pups was opened longitudinally and incubated for 15 min in Ca^2+^/Mg^2+^-free HBSS buffer supplemented with FCS (2%), HEPES (10 mM) and DTT (5 mM). Tissues were then transferred into a fresh Ca^2+^/Mg^2+^-free HBSS solution supplemented with FCS (2%), HEPES (10 mM) and EDTA (5 mM) and incubated for another 15 min before vortexing. The supernatant containing epithelial cells was subsequently filtered (100 μm) into cold FACS buffer. The last step was repeated for a total incubation time in EDTA buffer of 30 min. The epithelial fraction was kept on ice from this point onward. Tissue samples were moved to a Ca^2+^/Mg^2+^-containing HBSS solution supplemented with FCS (2%) and HEPES (10 mM) and incubated for another 10 min. Tissues were subsequently placed in Ca^2+^/Mg^2+^-containing HBSS medium supplemented with FCS (2%), HEPES (10 mM), Liberase (100 μg ml^−1^) and DNase I (30 μg ml^−1^), where they were manually cut into small pieces. Following a 20-min incubation, mechanical dissociation using gentleMACS C tubes (Miltenyi Biotec) and the program m_intestine_01 on the gentleMACS dissociator (Miltenyi Biotec) was performed. Samples were then filtered (100 μm) and kept on ice for subsequent staining. All incubations were performed at 37 °C under gentle agitation. For the lung samples, mouse lungs were perfused with cold PBS through the right ventricle and incubated in ice-cold RPMI 1640 until dissociation. Initial mechanical dissociation was performed using the m_lung_01_02 program on a gentleMACS dissociator (Miltenyi Biotec). Tissues were then digested with 50 µg ml^−1^ Liberase and 25 µg ml^−1^ DNase I (Roche) in prewarmed RPMI 1640 for 35 min at 37 °C with gentle rocking, followed by further dissociation using the m_lung_02_01 program. Single-cell suspensions were obtained by passing samples through 70-µm strainers. After red blood cell lysis (BD Pharm Lyse), cells were washed, filtered and stained for flow cytometry.

### Flow cytometry

For lamina propria immune cell staining, single-cell suspensions were incubated and blocked with anti-mouse CD16/CD32 antibody (BioLegend, 101340) diluted in FACS buffer (1× PBS, supplemented with 5% FCS and 0.5% sodium azide). The following antibodies, purchased from BD Horizon, Thermo Fisher Scientific or BioLegend, were used for surface molecule staining: CD45-BUV395 (564279), CD11b-BV510 (101263), CD11c-BV421 (117343), CD19-BV421 (115549), Ter119-BV421 (116234), NK1.1-BV421 (108741), CD49-PB (108918), CD8a-BV421 (100753), CD8a-BV510 (100752), CD3-PE-Cy7 (100220), CD4-BV650 (100555), KLRG1-PerCP-eFluor 710 (46-5893-82), huCD4-APC (300537), huCD4-PE (300539), Ly6G-AF700 (127622), Siglec-F-APC-Cy7 (155532), IL-17RB-APC (146308), CD90.2-BV785 (105331) and CD24-AF488 (101816). In the event of live cell acquisition, samples were resuspended in FACS buffer and immediately run on one of the three machines mentioned below. In case of continued intracellular staining, lamina propria cells were fixed for 10 min using the FOXP3/Transcription Factor Staining set (Thermo Fisher, 00-5523-00). Cells were then resuspended in 1× permeabilization buffer (Thermo Fisher, 00-8333-56) containing antibodies (GATA3-PE (12-9966-42), Ki-67-FITC (151212)) to intracellular targets. Cells were kept on ice throughout the staining procedure. For intestinal epithelial cell staining, cells were blocked with anti-mouse CD16/CD32 antibody. The following antibodies, purchased from BD Horizon or BioLegend, were used for surface staining: CD45-BUV395 (564279), CD24-PE-Cy7 (101822), Siglec-F-Alexa Fluor 647 (155520), CD326-PerCP-Cy5.5 (118220) and IL-17RB-APC (146308). The Zombie Red Fixable Viability Kit (BioLegend, 423110) was used to distinguish dead cells. Cells were fixed with 4% paraformaldehyde (Merck, 47608-250ml-f) and permeabilized using permeabilization buffer (Thermo Fisher, 00-8333-56). For intracellular staining, DCAMKL1 (Abcam, ab31704) was applied, followed by goat anti-rabbit IgG-AF488 (Thermo Fisher, A32731). Antibody dilutions are not specified, as they should be determined individually by each group based on the instrument and the number of cells used for staining. Multicolor flow cytometric analysis was performed using either the spectral analyzer Cytek Aurora with SpectroFlo software v3.1.0 (Cytek Biosciences) or the BD FACSymphony A5 with FACSDiva v9.1 software (BD Biosciences). Data were processed with FlowJo software v10 (BD Biosciences).

### Cell sorting

For sorting of ILC2s (defined as CD45^+^, Lin^−^ (here also including CD11b, CD8a and Siglec-F), CD3^–^, CD4^–^, Thy1.2^+^ and KLRG1^+^), roughly 10,000 cells were sorted into RLT lysis buffer (Qiagen) and pooled (2×) before RNA extraction and subsequent sequencing. For qPCR, tuft cells (CD45^low/mid^, EpCAM^+^, CD24^+^, *Il25*^tdTomato+^) and nontuft epithelial cells (CD45^–^, EpCAM^+^, *Il25*^tdTomato–^) were sorted directly into RLT lysis buffer (Qiagen). For proteome analysis, roughly 5,000 tuft cells (CD45^low/mid^, EpCAM^+^, CD24^+^, Flare25^+^) were sorted into SP3 lysis buffer (LC–MS water supplemented with HEPES (pH 8, 50 mM), SDS (1%), Triton X-100 (1%), IGEPAL CA-630 (1%), Tween 20 (1%), sodium deoxycholate (10 mg ml^−1^, prepared freshly on the day), NaCl (50 mM), EDTA (5 mM) and glycerol (1%)). Cell sorting was performed using the FACSymphony S6 cell sorter, and sorted samples were kept at −80 °C while awaiting further analysis.

### Sample processing for LC–MS

The samples were processed using S-Trap microspin columns (Protifi) according to the manufacturer’s protocols with some exceptions. The samples were thawed and sonicated for 5 min in an Elmasonic P60H bath sonicator (Elma Schmidbauer) at 100% and 37 kHz. The samples were then dried with an SPD120 SpeedVac (Thermo Fisher Scientific) set at 45 °C, resuspended in 23 µl of 1× lysis buffer (5% SDS, 50 mM TEAB, pH 8.5) and sonicated for 5 min in an Elmasonic P60H bath sonicator (Elma Schmidbauer) at 100% and 37 kHz. The samples were reduced with 1 µl of a reductant (final concentration 5 mM TCEP) at 55 °C for 15 min and alkylated by adding 1 µl of an alkylator (final concentration 20 mM chloroacetamide) at room temperature for 10 min. A 2.5-µl volume of the acidifier was added to the sample (final concentration ~2.5% phosphoric acid), and the tube was vortexed. Colloidal protein particulate was formed with the addition of 165 µl binding/wash buffer (90% aqueous methanol, 100 mM TEAB). The mixture was applied to an S-trap microcolumn in a 2-ml polypropylene receiver tube, and the column was centrifuged at 4,000*g* for 20 s to trap proteins. The columns were washed three times with 150 µl binding/wash buffer and centrifuged at 4,000*g* for 10 s with 180° rotation of the columns between washes. The S-Trap column was centrifuged for 1 min at 4,000*g* to remove the binding/wash buffer completely. The S-Trap microcolumn was transferred to a clean 2-ml polypropylene receiver tube, and digestion was performed with 20 µl digestion buffer containing 1 µg Pierce Trypsin/Lys-C Protease Mix overnight at 37 °C. The peptides were eluted with 40 µl of 50 mM TEAB followed by 40 µl of 0.2% aqueous formic acid and 40 µl of 50% acetonitrile. Peptides were finally dried with a SpeedVac SPD120 concentrator (Thermo Fisher Scientific) with no temperature applied. The peptides were resuspended in 25 µl of 3% acetonitrile and 0.1% formic acid. The peptide concentration was measured using the Pierce Quantitative Colorimetric Peptide Assay.

### LC–MS measurement

Samples were acquired on an EASY-nLC 1200 coupled to a Thermo Orbitrap Eclipse Tribrid mass spectrometer in data-independent acquisition mode. Buffer A was 0.1% formic acid in water, and buffer B was 0.1% formic acid in 80% acetonitrile. An Aurora Ultimate 25 × 75 C18 ultrahigh-performance LC column from IonOpticks was used in combination with a PepMap Neo C18 5 µm, 300 µm × 5 mm trap column from Thermo Fisher Scientific. The flow rate was set to 400 nl min^−1^, and the following gradient was applied: 30 s, 0–3% buffer B; 30 s, 3–6% buffer B; 27.5 min, 6–21% buffer B; 10.5 min, 21–31% buffer B; 6 min, 31–44% buffer B; 3 min, 44–100% buffer B; and a final wash for 7 min at 100% buffer B. For the MS1 scan, the Orbitrap resolution was set to 120,000, with quadrupole isolation turned on. A scan range of 380–980 *m*/*z* was applied, the RF lens was set to 30%, and a standard automatic gain control target with a custom maximum injection time of 50 ms was applied. For the MS2 scan, the Orbitrap resolution was set to 15,000, with 50 fixed windows (12 *m*/*z* isolation window and 0.5 *m*/*z* overlap). Higher-energy collisional dissociation collision energy was fixed at 30%. MS/MS scan range was defined as 145–1,450 *m*/*z*, and the RF lens was set to 30%. The automatic gain control target was set to 1,000% with a custom maximum injection time of 22 ms.

### Proteomics data analysis

Raw data were processed using DIA-NN software with default settings^59^. The search was performed library-free with an in silico digestion and deep learning-based spectra and retention time prediction. The mouse fasta file was downloaded from UniProt (UP000000589, downloaded on 15 July 2024). Downstream analysis was performed with R. For protein analysis, values normalized to the protein group were used. Precursors were filtered for proteotypicity, precursor *q* values were filtered with a threshold of 0.01, and protein group *q* values were filtered with a threshold of 0.05.

### Immunofluorescence

A 4- to 6-cm piece of proximal small intestinal tissue was flushed with PBS, cut open longitudinally and fixed for >2 h in paraformaldehyde (4% w/v) at 4 °C. This was followed by a PBS wash and an overnight incubation in sucrose (30% w/v) at 4 °C. Folded into ‘swiss-rolls’, tissues were subsequently embedded in OCT and stored at −80 °C before sectioning (6 μm) on a Leica CM1850 cryostat (Leica Biosystems). Sections were incubated for 1 h at room temperature in blocking buffer (1× PBS supplemented with 2% BSA, 0.1% Triton X-100 and 5% goat serum). All staining was subsequently performed in blocking buffer. Incubation with the primary antibody (DCLK1 (Abcam, ab31704, 1:1,000) or MUC2 (Santa Cruz Biotechnology, sc-15334, 1:100)) was performed at 4 °C overnight, and incubation with the secondary antibody (goat anti-rabbit IgG-AF488 (Thermo Fisher, A32731, 1:2,000)) was performed for 1 h at room temperature. DAPI was added separately for 5 min, and samples were washed with PBS. For mounting, Vectashield mounting medium was diluted 1:3 in a glycerol solution with added Tris (50 mM). Images were acquired on a fluorescence microscope (20× Leica DMi8 Thunder Imager) with LAS X software 4.7.0 (Leica). Fiji v1.53 (ImageJ) software was used to analyze and process the images. For goblet cell analysis, grayscale images of MUC2 and DAPI immunofluorescence staining were processed with Fiji v1.53. Automatic brightness adjustment was performed, followed by the application of Otsu’s automatic thresholding algorithm and measurement of the MUC2 and DAPI areas.

### RNA sequencing

RNA was isolated from FACSorted samples using the Quick-RNA Microprep kit (Zymo Research, R1050). For RNA sequencing, samples were then stored for <1 week at −80 °C before being shipped on dry ice to Novogene. Samples underwent SMARTer amplification before being sequenced on an Illumina platform, where paired-end reads were generated. We estimated transcript counts using Kallisto v0.48.0 (ref. ^60^) with the mm10 reference genome and used tximport v1.28.0 (ref. ^61^) to import them in R v4.3.2. Based on normalized counts, we removed three outlier samples using the PcaProj function from the rrcov v1.7.4 (ref. ^62^) R package and proceeded with differential expression analysis using DESeq2 v1.40.1 (ref. ^63^). Normalized counts from DESeq2 for the selected genes were used to generate heatmaps with ComplexHeatmap v2.16.0 (ref. ^64^).

### Reverse transcription followed by qPCR

RNA was isolated from FACSorted samples using the Quick-RNA Microprep kit (Zymo Research, R1050). Samples were processed further using the SuperScript IV VILO Master Mix with ezDNase Enzyme (Thermo Fisher Scientific, 11766050). The resulting cDNA was then mixed with PowerUp SYBR Green Master Mix (Thermo Fisher Scientific, A25918) and run on a 7500 Fast Real-Time PCR system (Applied Biosystems). Transcripts were normalized to *Rps17* (40S ribosomal protein S17), and relative expression is shown as 2^−ΔCt^. The primer sequences were as follows: *Rps17*, 5′-CGCCATTATCCCCAGCAAG-3′, 5′-TGTCGGGATCCACCTCAATG-3′; *Il25*, 5′-ACAGGGACTTGAATCGGGTC-3′, 5′-TGGTAAAGTGGGACGGAGTTG-3′; *Dclk1*: 5′-CAGCCTGGACGAGCTGGTGG-3′, 5′-TGACCAGTTGGGGTTCACAT-3′; and *Il17rb*, 5′-GGCTGCCTAAACCACGTAATG-3′, 5′-CCCGTTGAATGAGAATCGTGT-3′.

### Confocal Ca^2+^ imaging of tuft cells in an en-face ileum and scraped villus preparation

Mice (6–22 weeks old, both sexes) were decapitated after CO_2_ anesthesia. The intestine was excised and cleansed by perfusing with an extracellular solution containing 136.5 mM NaCl, 5.6 mM KCl, 2.2 mM CaCl_2_, 1 mM MgCl_2_ and 10 mM HEPES adjusted to pH 7.4 (NaOH) and 290 mOsm (~10 mM glucose). The ileum, approximately the last quarter of the intestine, was divided into four sections and placed on small strips of tissue paper (Kimtech Science) to prevent the en-face ileum from curling after cutting the tube-like structure open. The four en-face ileum parts were then placed into separate recording chambers (Luigs & Neumann) and secured using a tissue slice holder (also called a ‘harp’) to prevent movement caused by the speed of the solution flow through the chamber^65,66^. In the case of the scraped villus preparation, the whole ileum was scraped using a scalpel blade no. 10. The scraped villi were directly added to a solution containing the Ca^2+^ indicator, slightly triturated and placed into the recording chamber containing an acid-washed glass coverslip coated twice with 0.01% poly(l-lysine) (Sigma P-6282, CAS 25988-63-0). The scraped villi were also secured using a tissue slice holder (harp) to prevent movement.

Intracellular Ca^2+^ was monitored with either GCaMP6f expressed in the tuft cells of *Trpm5*^Cre^; *R26*^GcaMP6f^ mice or the Ca^2+^ indicator Cal-630 AM loaded into small intestinal epithelial cells using a similar technique as described previously for the tracheal epithelium^22^. Cal-630 AM (AAT Bioquest, cat. no. 20720) was dissolved in a solution of DMSO and freshly prepared 20% Pluronic F-127 in DMSO; then, the solution was further diluted in the extracellular solution (see above) and briefly sonicated. The en-face ileum or scraped villi were subsequently incubated in the Cal-630 AM loading solution with a final concentration of 4.1 μM Cal-630 AM, 0.15% DMSO and 0.01% Pluronic F-127 for 90–120 min at room temperature. Imaging experiments were performed using an upright confocal laser scanning microscope (Zeiss LSM 880 Indimo) with a Plan-Apochromat 20×/1.0 water immersion objective. The excitation wavelength for GCaMP6f was 488 nm, and emitted fluorescence was collected between 500 and 540 nm. For calcium imaging in IP3R2 mice, the tuft cells were identified using an excitation wavelength for tdTomato of 561 nm and an emission wavelength between 565 and 593 nm. The excitation wavelength for the Ca^2+^ indicator Cal-630 was 594 nm, and emitted fluorescence was collected between 600 and 690 nm. The excitation wavelength used for the Ca^2+^ indicator Cal-630 is unable to excite the tdTomato fluorescence. To prevent Cal-630 emissions from being collected due to the possibility of exciting the indicator with the 561-nm laser, we limited the emission wavelength for tdTomato to a maximum of 594 nm. All scanning head settings were kept constant during each experiment. Optical sections had a thickness of 12.1 μm and were kept constant in all recordings. Images (512 × 512 pixels per frame) were acquired every 0.631 s. The following criteria for stimulus-induced Ca^2+^ responses were applied: (1) a response was defined as a stimulus-dependent deviation of either a GCaMP6f or Cal-630 fluorescence signal that exceeded twice the s.d. of the mean of the baseline fluorescence noise; (2) a response had to occur within 2 min after stimulus application. In time series experiments, ligand application was repeated to confirm the repeatability of a given Ca^2+^ response. GCaMP6f or Cal-630 fluorescence changes of individual cells are expressed as relative fluorescence changes (that is, Δ*F*/*F*, where *F* is the average fluorescence during control stimulation with the extracellular solution). The maximum change in relative fluorescence is indicated as the *F*_peak_. Images were processed with ZenBlack software (Zeiss) and analyzed using Fiji/ImageJ (National Institutes of Health), Igor Pro 6.12 (WaveMetrics) and Origin 7.5 (OriginLab) software. Through the Igor Pro software package, user-defined functions in combination with an iterative Levenberg–Marquardt nonlinear, least-squares fitting routine were applied to the data. Dose–response curves were fitted by the following equation:

$${f(x)}={E_{\rm{min}}} +(({E_{\rm{max}}}-{E_{\rm{min}}})){ / }\left(\left\{{1+[{\rm{EC}}_{50}/{{x}}]}^{{{n}}}\right\}\right)$$

where *x* is the drug concentration, *E*_min_ is the baseline response, *E*_max_ is the maximal response at saturating concentrations and EC_50_ is the drug concentration that produces 50% of the maximal response, with slope *n* being the Hill coefficient of the sigmoid curve.

The intestinal epithelium was stimulated successively using bath application. Chemostimuli for Ca^2+^ imaging were prepared fresh daily and diluted in the extracellular solution. The final succinate (CAS 6106-21-4) concentrations were 0.3, 1.0, 3.0 and 10.0 mM. The impact of intracellular Ca^2+^ stores was examined using CPA (10 μM, CAS 18172-33-3). Final DMSO concentrations (<0.1%, vol/vol) were tested in control solutions and had no effects. All chemicals were obtained from Merck (previously Sigma-Aldrich) unless otherwise stated. The extracellular 60 mM KCl solution contained 82.1 mM NaCl, 60 mM KCl, 2.2 mM CaCl_2_, 1 mM MgCl_2_ and 10 mM HEPES adjusted to pH 7.4 (NaOH) and 290 mOsm (~10 mM glucose).

### 3D and 2D organoid culture and stimulation

Mouse duodenal tissue was flushed with cold PBS, opened longitudinally and scraped with a glass coverslip to remove the villi. The remaining tissue was placed into 10 ml PBS (no Ca^2+^/Mg^2+^) supplemented with EDTA (5 mM), DTT (5 mM) and penicillin–streptomycin (PenStrep, 1×; Thermo Fisher Scientific, 15140148). Following a 10-min incubation, the tissue was transferred to 10 ml PBS (no Ca^2+^/Mg^2+^) supplemented with EDTA (3 mM), DTT (2 mM) and PenStrep (1×) for another 10-min incubation. Both incubations took place at 4 °C with gentle shaking of the sample. The tissue was then transferred to 20 ml DMEM F12 (Thermo Fisher Scientific, 10565018) with PenStrep (1×) and shaken vigorously for 1 min. The crypt-rich supernatant was passed through a 100-µm filter and spun down at 300*g* at 4 °C for 3 min. The resulting pellet was resuspended in prewarmed ENR medium consisting of DMEM F12, R-spondin (produced in-house; Bio-Techne, 3710-001-01), PenStrep (1×), HEPES (10 mM), *N*-acetylcysteine (500 mM), EGF (100 µg ml^−1^, Peprotech), Noggin (50 ng ml^−1^, U-Protein Express), N2 supplement (1×, Thermo Fisher Scientific, 17502048) and B27 supplement (1×, Thermo Fisher Scientific, 17504044). For 3D organoid cultures, the suspension was mixed 1:1 with Matrigel (Corning, CLS356231) and plated into small domes (40 µl) containing 500 crypts each. ENR medium was added to cover the domes and subsequently exchanged every 3–4 days. Organoid cultures were passaged every 7 days and maintained for a maximum of 10 weeks. Stimulation was performed with the indicated concentrations of cytokines. For 2D organoid cultures, a 24-well plate was precoated with 2% Matrigel in ENR medium supplied with A83-01 (500 nM) and Y-27632 (10 μM), followed by plating of 1,000 crypts per well. For the stimulation of 2D organoids, the medium was gently aspirated 22 h after plating the crypts. The organoids were washed once with warm PBS and added with 300 µl of ENR medium. After 1 h, the ENR medium was gently aspirated and 150 μl RPMI 1640 medium containing either DMSO, ionomycin (1 μg ml^−1^) or dibasic sodium succinate hexahydrate (50 mM) was added. After stimulation for 30 min at 37 °C, the supernatant from each well was collected and IL-25 was quantified by ELISA (BioLegend, 447104) according to the manufacturer’s instructions.

### Statistics and reproducibility

Unless otherwise indicated, data are pooled from two or more repeat experiments and displayed as the mean values (±s.e.m.) in all graphs, with *n* reflecting biological replicates. For statistical analysis of data generated from two groups, a two-tailed (unpaired, unless otherwise indicated in the figure legend) *t* test was used when a normal distribution could be assumed; otherwise, the Mann–Whitney *U* test was used. For statistical analysis of more than two groups, an ordinary one-way ANOVA with Tukey’s multiple-comparison test was used throughout, apart from a two-way ANOVA being used for data containing two independent variables (see the legend of Fig. 3). Results were deemed significant when *P* < 0.05. Except in the case of major experimental errors, no data were excluded from the analysis. Intragroup variation was not assessed. All statistical analyses, except for data generated in RNA sequencing, were performed using Prism 10 (GraphPad Software) or Origin Pro (OriginLab).

The number of experiment repeats is detailed in what follows. Figure 1: a–d, two independent experiments; e–g, two independent experiments; h,i, two independent experiments; j, two independent experiments. Figure 2: a–c, two independent experiments; d, one experiment; e–o, each tuft cell measurement is an independent experiment. Figure 3: a,b, two independent experiments; c,d, two independent experiments; e,f, three independent experiments; h,i, one experiment representative of three independent experiments. Figure 4: a,b, two independent experiments; c, two independent experiments; d, two independent experiments; e, two independent experiments; f–h, four independent experiments; i, one experiment; j,k, two independent experiments; l–n, two independent experiments. Figure 5: a,b, two independent experiments; c, two independent experiments; d,e, two independent experiments; f–i, two independent experiments; j–m, two independent experiments. Figure 6: a,b, two independent experiments; c,d, two independent experiments; e–g, two independent experiments; h, two independent experiments; i, two independent experiments; k,i, three independent experiments. Figure 7: a,b, three independent experiments; c,d, three independent experiments; e–g, two independent experiments; h, two independent experiments; i, two independent experiments; k,l, two independent experiments. Figure 8: b,c, two independent experiments; e,f, two independent experiments; g, two independent experiments; h,i, three independent experiments; j–l, two independent experiments; n, two independent experiments. Extended Data Fig. 1: a–e, two independent experiments; f, two independent experiments; g,h, four independent experiments; i, two independent experiments. Extended Data Fig. 2: all experiments were repeated two or more times. Extended Data Fig. 3: a–c, two independent experiments; d,e, two independent experiments. Extended Data Fig. 4: a,b, four independent experiments; c, one experiment; d,e, two independent experiments; f, three independent experiments; g, two independent experiments; h–j, two independent experiments; k,l, two independent experiments; m, one representative data from two independent experiments; n, three independent experiments; o, two independent experiments; p, two independent experiments; q, two independent experiments; r, one experiment. Extended Data Fig. 5: a, two independent experiments; b, two independent experiments; c, two independent experiments; d,e, two independent experiments. Extended Data Fig. 6: a,b, two independent experiments; c, two independent experiments.

### Reporting summary

Further information on research design is available in the Nature Portfolio Reporting Summary linked to this article.

## Online content

Any methods, additional references, Nature Portfolio reporting summaries, source data, extended data, supplementary information, acknowledgements, peer review information; details of author contributions and competing interests; and statements of data and code availability are available at 10.1038/s41590-025-02104-y.

## Supplementary information

Reporting Summary

Supplementary Video 1 Example of succinate-induced intercellular Ca^2+^ responses in *Trpm5*^Cre^; *R26*^GCaMP6f^ tuft cells of the ileum from an ex vivo whole-mount (en face view of the ileum) preparation from *Trpm5*^Cre^; *R26*^GCaMP6f^ mice. The speed of the video was increased by 10×. Data are representative of multiple independent experiments.

## Data Availability

The RNA-sequencing data were submitted to the National Center for Biotechnology Information’s Gene Expression Omnibus under accession number GSE279898. The proteomics raw and processed datasets have been deposited at Mendeley Data (10.17632/hkfn5gy5vs.1).
