# Supplementary material for: Tuft cell IL-17RB restrains IL-25 bioavailability and reveals context-dependent ILC2 hypoproliferation
Source: Nat Immunol. 2025 Mar 12;26(4):567–81. doi: 10.1038/s41590-025-02104-y (PMC11957993; doi:10.1038/s41590-025-02104-y)
Supplement: Supplementary file 1 — Reporting Summary [file 41590_2025_2104_MOESM1_ESM.pdf]

nature portfolio

Corresponding author(s): Christoph Schneider

Last updated by author(s): Jan 23, 2025

Reporting Summary

Nature Portfolio wishes to improve the reproducibility of the work that we publish. This form provides structure for consistency and transparency in reporting. For further information on Nature Portfolio policies, see our Editorial Policies and the Editorial Policy Checklist.

Statistics

For all statistical analyses, confirm that the following items are present in the figure legend, table legend, main text, or Methods section.

n/a	Confirmed	
☐	☒	The exact sample size (*n*) for each experimental group/condition, given as a discrete number and unit of measurement
☐	☒	A statement on whether measurements were taken from distinct samples or whether the same sample was measured repeatedly
☐	☒	The statistical test(s) used AND whether they are one- or two-sided<br>*Only common tests should be described solely by name; describe more complex techniques in the Methods section.*
☒	☐	A description of all covariates tested
☐	☒	A description of any assumptions or corrections, such as tests of normality and adjustment for multiple comparisons
☐	☒	A full description of the statistical parameters including central tendency (e.g. means) or other basic estimates (e.g. regression coefficient) AND variation (e.g. standard deviation) or associated estimates of uncertainty (e.g. confidence intervals)
☐	☒	For null hypothesis testing, the test statistic (e.g. *F*, *t*, *r*) with confidence intervals, effect sizes, degrees of freedom and *P* value noted<br>*Give P values as exact values whenever suitable.*
☒	☐	For Bayesian analysis, information on the choice of priors and Markov chain Monte Carlo settings
☒	☐	For hierarchical and complex designs, identification of the appropriate level for tests and full reporting of outcomes
☒	☐	Estimates of effect sizes (e.g. Cohen's *d*, Pearson's *r*), indicating how they were calculated

Our web collection on statistics for biologists contains articles on many of the points above.

Software and code

Policy information about availability of computer code

Data collection	Flow cytometry data were collected using FACS Diva Software v9.1 or SpectroFlo® Software version 3.1.0.<br>Microscopy images were acquired using LAS X software 4.7.0 (Leica).<br>RNA sequencing samples underwent SMARTer amplification, prior to sequencing on an Illumina platform.<br>Proteomics data was acquired using EASY-nLC1200 coupled to a Thermo Orbitrap Eclipse Tribrid MS in data-independent acquisition (DIA) mode.
Data analysis	FlowJo 10.6.2 and 10.8.1 (Tree star) was used for the Flow cytometry data analysis.<br>GraphPad Prism v 7.03 and 9.4.0 (Graphpad Software Inc.) was used to prepare graphs and perform statistical analysis.<br>LAS X (Leica) software, and Fiji (Image J) software was used for image analysis. Fiji version 1.53 (Image J), Igor Pro 6.12 (Wavemetrics) and Originlab (Origin 7.5) software was used to analyze and process the images.<br>R and R studio v3.2-v4.0 was used for bioinformatics analysis of RNA sequencing results, detailed in the Method section<br>Proteomics data: Raw data was processed using DIA-NN software with default settings 59. Search was performed library-free with an in-silico digestion and deep learning–based spectra and retention time prediction.

For manuscripts utilizing custom algorithms or software that are central to the research but not yet described in published literature, software must be made available to editors and reviewers. We strongly encourage code deposition in a community repository (e.g. GitHub). See the Nature Portfolio guidelines for submitting code & software for further information.

Data

Policy information about availability of data

All manuscripts must include a data availability statement. This statement should provide the following information, where applicable:

- Accession codes, unique identifiers, or web links for publicly available datasets
- A description of any restrictions on data availability
- For clinical datasets or third party data, please ensure that the statement adheres to our policy

The RNA-seq data were submitted to the NCBI Gene Expression Omnibus under accession number GSE279898. The proteomics raw and processed dataset have been deposited at Mendeley Data (doi: 10.17632/hkfn5gy5vs.1).
For RNA-seq, mm10 reference genome was used. For proteomics, mouse fasta file was downloaded from UNIPROT (UP000000589, downloaded on 15 July 2024).

Research involving human participants, their data, or biological material

Policy information about studies with human participants or human data. See also policy information about sex, gender (identity/presentation), and sexual orientation and race, ethnicity and racism.

Reporting on sex and gender	N/A
Reporting on race, ethnicity, or other socially relevant groupings	N/A
Population characteristics	N/A
Recruitment	N/A
Ethics oversight	N/A

Note that full information on the approval of the study protocol must also be provided in the manuscript.

Field-specific reporting

Please select the one below that is the best fit for your research. If you are not sure, read the appropriate sections before making your selection.

☒ Life sciences ☐ Behavioural & social sciences ☐ Ecological, evolutionary & environmental sciences

For a reference copy of the document with all sections, see nature.com/documents/nr-reporting-summary-flat.pdf

Life sciences study design

All studies must disclose on these points even when the disclosure is negative.

Sample size	Sample sizes were chosen based on the principle of 3Rs. The smallest sample size was chosen that could give a significant difference. Given the robustness of the phenotypes across all methods used, the minimum sample size assuming no overlap in control versus experimental is three animals per experiment.
Data exclusions	Based on normalized counts we removed 3 outlier samples using PcaProj function from the rrcov v1.7.4 R package during RNA sequencing analysis, as described in the Method section. Other than that, no samples were excluded from analysis unless in case of major experimental error (sample dropped on floor, re-genotyping revealed incorrect use of animal etc.). When FACS data is displayed, pre-gating is always annotated in the respective figure legends, and often also in the graphs themselves.
Replication	Unless otherwise noted in the Figure legend (e.g. for RNA sequencing), experiments were independently repeated two or more times, with the number of repeats annotated in each respective figure legend.
Randomization	All experiments were performed on male and female mice, without separation between experimental groups and controls. Across methods, samples were analyzed equally without sub-sampling, hence no randomization was required.
Blinding	As the same person was often responsible for genotyping, experimental treatments, tissue harvest and data analysis, it was not possible to completely blind the investigator. However, when possible, little or no attention was given to experimental group allocation before data analysis.

Reporting for specific materials, systems and methods

We require information from authors about some types of materials, experimental systems and methods used in many studies. Here, indicate whether each material, system or method listed is relevant to your study. If you are not sure if a list item applies to your research, read the appropriate section before selecting a response.

Materials & experimental systems

n/a	Involved in the study
☐	☒ Antibodies
☒	☐ Eukaryotic cell lines
☒	☐ Palaeontology and archaeology
☐	☒ Animals and other organisms
☒	☐ Clinical data
☒	☐ Dual use research of concern
☒	☐ Plants

Methods

n/a	Involved in the study
☒	☐ ChIP-seq
☐	☒ Flow cytometry
☒	☐ MRI-based neuroimaging

Antibodies

Antibodies used	For flow cytometry: Anti-mouse CD45, BUV395 (30-F11) BD Horizon 564279; Anti-mouse CD11b, BV510(M1/70) 101263; Anti-mouse CD11c, BV421 (N418) BioLegend 117343; Anti-mouse CD19, BV421 (6D5) BioLegend 115549; Anti-mouse Ter119, BV421 (TER-119) BioLegend 116234; Anti-mouse NK1.1, BV421 (PK136) BioLegend 108741; Anti-mouse CD49b, PB (DX5) BioLegend 108918; Anti-mouse CD8a, BV510 (53-6.7) BioLegend 100752; Anti-mouse CD4, BV650 (RM4-5) BioLegend 100555; Anti-mouse CD11b, BV711 (M1/70) BioLegend 101241; Anti-mouse Ki-67, FITC (11F6) BioLegend 151212; Anti-mouse KLRG1, PerCPeFluor710 (2F1) Thermo Fisher 46-5893-82; Anti-mouse Gata3, PE (TWAJ) Thermo Fisher 12-9966-42; Anti-human CD4, APC (RPA-T4) BioLegend 300537; Anti-mouse Ly6G, AF700 (1A8 BioLegend 127622; Anti-mouse SiglecF, APC-Cy7 (S17007L) BioLegend 155532; Anti-mouse IL-17RB, APC (9B10) (used 1:20) BioLegend 146308; Anti-mouse Thy 1.2, BV785 (30-H12) BioLegend 105331; Anti-mouse CD326, PerCP-Cy5.5 (G8.8) BioLegend 118220; Anti-mouse CD24, PE-Cy7 (M1/69) BioLegend 101822; Anti-mouse CD16/32 (93) BioLegend 101340; Anti-mouse Siglec-F, Alexa Fluor 647 (S17007L) BioLegend 155520; Anti-mouse CD8a, BV421 (53-6.7) BioLegend 100753; Anti-mouse Gata3, AF488 (TWAJ) Thermo Fisher 53-9966-42; Anti-mouse CD3, PE-Cy7 (17A2) BioLegend 100220; Anti-human CD4, PE (RPA-T4) BioLegend 300508; Rabbit anti-mouse DCAMKL1 Abcam ab31704; anti-HA rat IgG1 (3F10) Sigma 11867423001; Goat anti-rabbit IgG (AF488) Thermo Fisher A32731; DAPI Merck 10236276001; Zombie Red BioLegend 423110.<br>Antibody dilutions are not specified, as they should be determined individually by each group based on the instrument and the number of cells used for staining.<br><br>For IF: DAPI Merck 10236276001; Rabbit anti-mouse MUC2 Santa Cruz sc-15334 (1:100); Rabbit anti-mouse DCAMKL1 Abcam ab31704 (1:1000); Goat anti-rabbit IgG (AF488) Thermo Fisher A32731 (1:2000).<br><br>Monoclonal blocking antibody against IL-17RB (Clone D9.2) was purified in house and administered i.p. at the indicated time points (antibody injection dose: adult mice, 200 ug; pups, 50 ug).
Validation	All antibodies have been previously validated extensively by the manufacturer including by flow cytometry and IF, and confirmed by the authors for specificity and localization (no primary antibody control for IF and FMO for flow cytometry).

Animals and other research organisms

Policy information about studies involving animals; ARRIVE guidelines recommended for reporting animal research, and Sex and Gender in Research

Laboratory animals	We used the following mouse strains: Vil1Cre (B6.Cg-Tg(Vil1-cre)997Gum/J; JAX, 004586); Vil1CreERT2, Nmur1iCre-eGFP; Il17rbfl/fl; Il17rb–/– mice with global Il17rb deletion were generated from Vil1Cre; Il17rbfl/fl mice in which Cre is active with high frequency in the male germline; Itpr2–/–; Trpm5Cre;R26GCaMP6f; Il17rc–/– ; Il5Red5 (B6(C)-Il5tm1.1(iCre)Lky/J; JAX, 030926); Il25fl-tdTomato, B6(C)-Il25tm1.1Lky/J); Il25iCre, B6(C)-Il25tm2.1(cre)Lky/J); TgCAG-lsl-Gq-DREADD (B6N;129-Tg(CAG-CHRM3*,-mCitrine)1Ute/J; JAX, 026220). B6(C)-Gt(ROSA)26Sortm1(Nfkb-destEGFP)Kopf. Except for Trpm5Cre;R26GcaMP6f and NF-κB-reporter mice, all lines were crossed to Arg1Yarg;Il13Smart13 double reporter (B6.129S4-Arg1tm1Lky/J; JAX, 015857; B6.129S4(C)-Il13tm2.1Lky/J; JAX, 031367). All mice were on a C57BL/6 background. Animals aged between 7 days and 16 weeks were used. Mice were maintained in a specific-pathogen-free (SPF)facility with a 12-h light-dark cycle, under controlled temperature (18-23°C) and humidity (40-60%), with ad libitum standard diet and water.
Wild animals	No wild animals were used in the study.
Reporting on sex	All experiments included both male and female mice, equally distributed throughout the experimental groups. Data was initially analyzed separated by sex, but no meaningful differences could ever be found. We therefore proceeded with the paper without separating sexes, not during the experiment, and not during analysis.
Field-collected samples	No field-collected samples were used in the study.
Ethics oversight	Animal experiments were reviewed and approved by the cantonal veterinary office of Zurich (permit numbers 054/2022 and 111/2022), and in accordance with the guidelines established by the German Animal Welfare Act, European Communities Council Directive 2010/63/EU, the institutional ethical and animal welfare guidelines of Saarland University (approval number of the Institutional Animal Care and Use Committee: CIPMM-2.2.4.1.1).

Note that full information on the approval of the study protocol must also be provided in the manuscript.

Plants

Seed stocks	N/A
Novel plant genotypes	N/A
Authentication	N/A

Flow Cytometry

Plots

Confirm that:

☒ The axis labels state the marker and fluorochrome used (e.g. CD4-FITC).

☒ The axis scales are clearly visible. Include numbers along axes only for bottom left plot of group (a 'group' is an analysis of identical markers).

☒ All plots are contour plots with outliers or pseudocolor plots.

☒ A numerical value for number of cells or percentage (with statistics) is provided.

Methodology

Sample preparation	Flow cytometry: Adult mice were euthanized through carbon dioxide, and pups by decapitation. duodenal tissue from adult mice, or complete intestine from pups, was opened longitudinally and incubated for 15 minutes in Ca2+/Mg2+ -free HBSS buffer supplemented with FCS (2 %), HEPES (10 mM) and DTT (5 mM). Tissues were then transferred into a fresh Ca2+/Mg2+ -free HBSS solution supplemented with FCS (2 %), HEPES (10 mM), EDTA (5 mM), and incubated for another 15 minutes prior to vortexing. The supernatant containing epithelial cells, was subsequently filtered (100 m) into cold FACS buffer. The last step was repeated for a total incubation time in EDTA-buffer of 30 minutes. The epithelial fraction was kept on ice from this point on. Tissue samples were moved to a Ca2+/Mg2+ -containing HBSS solution supplemented with FCS (2 %), HEPES (10 mM), and incubated for another 10 minutes. Tissues were subsequently placed in Ca2+/Mg2+ -containing HBSS media supplemented with FCS (2 %), HEPES (10 mM), Liberase TM (100 ug/mL) and DNase 1 (30 ug/mL), where they were manually cut into small pieces. Following a 20-minute incubation, mechanical disassociation using GentleMACS C tubes (Miltenyi Biotec) and program m_intestine_01 on the gentleMACS dissociator (Miltenyi Biotec) was employed. Samples were then filtered (100 um) and kept on ice for subsequent staining. All incubations were performed at 37 C under gentle agitation.
Instrument	Flow cytometry analysis was performed on the BD FACSymphony and/or Cytek Aurora. Samples for RNAseq were sorted on a BD instrument S6 cell sorter.
Software	FlowJo 10.6.2 and 10.8.1 (Tree star), BD FACS DIVA and SpectroFlo® Software.
Cell population abundance	ILC2s were sorted based on widely accepted markers. Samples were re-sorted before actual acquisition to verify that they fell into the correct gate.
Gating strategy	Events were initially gated by FSC-A and SSC-A, then by FSC-A and FSC-H (to exclude doublets). Live CD45+ cells were then gated using a viability dye. Subsequent gating depends on the population of interest and is outlined in Supplementary Information.

☒ Tick this box to confirm that a figure exemplifying the gating strategy is provided in the Supplementary Information.
